# Facile route to synthesize Fe_3_O_4_@acacia–SO_3_H nanocomposite as a heterogeneous magnetic system for catalytic applications†

**DOI:** 10.1039/d0ra07986c

**Published:** 2020-11-04

**Authors:** Reza Taheri-Ledari, Mir Saeed Esmaeili, Zahra Varzi, Reza Eivazzadeh-Keihan, Ali Maleki, Ahmed Esmail Shalan

**Affiliations:** Catalysts and Organic Synthesis Research Laboratory, Department of Chemistry, Iran University of Science and Technology (IUST) Tehran 16846-13114 Iran maleki@iust.ac.ir +98 21 73021584 +98 21 77240640-50; Central Metallurgical Research and Development Institute (CMRDI) P. O. Box 87 Helwan Cairo 11421 Egypt a.shalan133@gmail.com; BCMaterials, Basque Center for Materials, Applications and Nanostructures Martina Casiano, UPV/EHU Science Park, Barrio Sarriena s/n Leioa 48940 Spain

## Abstract

In this work, a novel catalytic system for facilitating the organic multicomponent synthesis of 9-phenyl hexahydroacridine pharmaceutical derivatives is reported. Concisely, this catalyst was constructed from acacia gum (gum arabic) as a natural polymeric base, iron oxide magnetic nanoparticles (Fe_3_O_4_ NPs), and sulfone functional groups on the surface as the main active catalytic sites. Herein, a convenient preparation method for this nanoscale composite is introduced. Then, essential characterization methods such as various spectroscopic analyses and electron microscopy (EM) were performed on the fabricated nano-powder. The thermal stability and magnetic properties were also precisely monitored *via* thermogravimetric analysis (TGA) and vibrating-sample magnetometry (VSM) methods. Then, the performance of the presented catalytic system (Fe_3_O_4_@acacia–SO_3_H) was further investigated in the referred organic reaction by using various derivatives of the components involved in the reaction. Optimization, mechanistic studies, and reusability screening were carried out for this efficient catalyst as well. Overall, remarkable reaction yields (94%) were obtained for the various produced derivatives of 9-phenyl hexahydroacridine in the indicated optimal conditions.

## Abstract 2

We designed and fabricated a novel catalytic system with high heterogeneity and magnetic features to facilitate the MCR synthetic reactions of 9-phenyl hexahydroacridine pharmaceutical derivatives.

## Graphical abstract

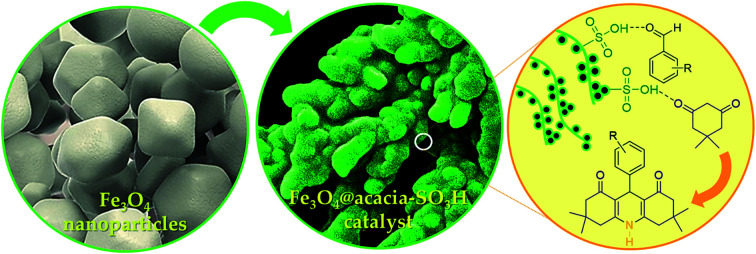

## 1. Introduction

Recently, powder technology has received much attention in heterogeneous catalytic systems; powders have great potential to be applied in complex organic synthesis reactions and are conveniently separated during the purification processes through their heterogeneity.^1,2^ One of the best-known members of this family of materials is magnetic nanoscale powder, which has been widely used for various scientific purposes such as drug delivery,^3^ disease diagnosis,^4^ water desalination,^5^ environment cleaning,^6^ and chemical catalysis.^7^ In our previous work, we have reported several different catalytic systems constructed with the individual iron oxide (Fe_3_O_4_) powder (in nanoscale) and applied them in various catalytic processes.^8–15^ These nanoparticles could also be composed of other fibrous materials and could be immobilized into polymeric matrices. Through this method, the general properties of the catalytic systems such as the physicomechanical features of the individual Fe_3_O_4_ powder are improved. In this regard, numerous studies have been performed, and it has been revealed that the efficiency of the Fe_3_O_4_ powder can be significantly modified through its composition with other materials.^16–20^ For instance, a composition of graphene oxide, Fe_3_O_4_ and silver nanoparticles was prepared and applied for enhanced photocatalytic degradation of phenols in the past year.^21^ Moreover, Javanbakht *et al.* composited magnetic nanoparticles with a chitosan matrix for the efficient removal of lead(ii) from water resources.^22^ Here, we attempted to prepare a suitable composite of Fe_3_O_4_ nanoparticles and “acacia gum” powder, and we applied this composition to facilitate the organic synthesis of 9-phenyl hexahydroacridine (HHA) pharmaceuticals.

**Fig. 1:**
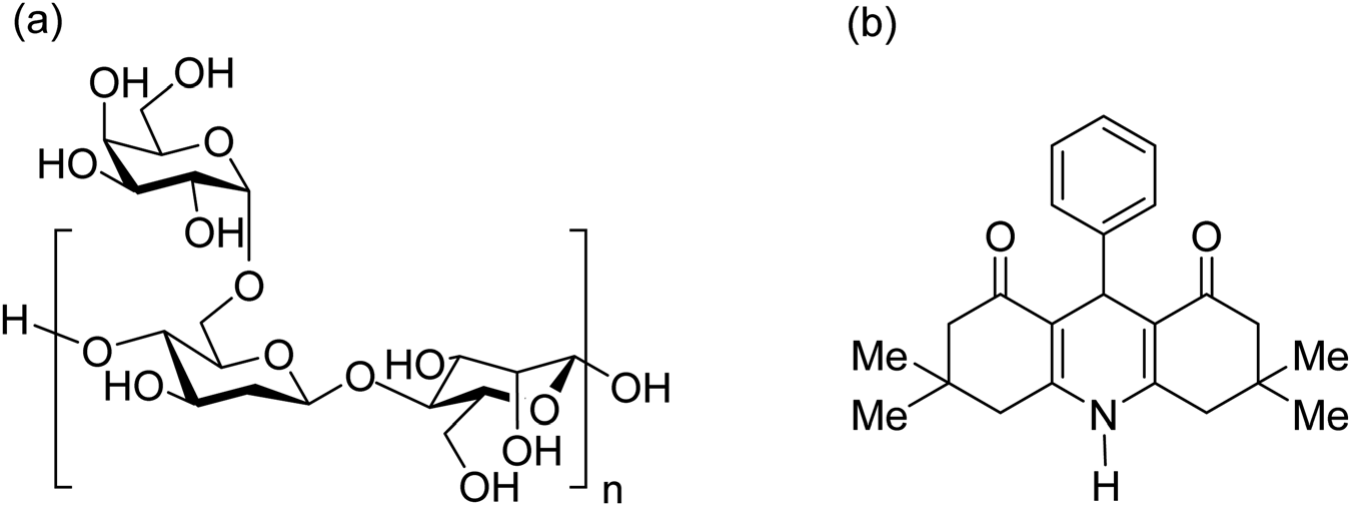
(a) Chemical structure of the acacia gum polymer and (b) general structure of tetramethyl-9-phenyl-hexahydroacridine-1,8(2*H*,5*H*)-dione.

Acacia gum, also called “gum arabic”, is obtained from wild trees, and its main origin is Somalia. From the organic chemical aspect, there are several hydroxyl groups in the structure of this polymer that can be used as appropriate sites for covalent binding and catalytic applications.^23^ From the physicomechanical aspect, the suitable stability of acacia gum led us to apply it as an appropriate substrate for immobilization of magnetic nanoparticles. Previously, acacia gum was used as a matrix for catalytic systems. For instance, Banerjee and Chen used this polymer to design a nanoscale absorbent system for the removal of copper ions from water resources. They also magnetized acacia gum through the composition of Fe_3_O_4_ NPs for an easy separation from the mixture.^24^ In this work, we attempted to perform a chemical modification of acacia gum by sulfonation of the hydroxyl functional groups and use the product as an acidic catalytic system for the facilitated synthesis of HHA derivatives. The chemical structure of the acacia gum polymer is presented in Fig. 1(a).

To date, various types of hydroacridine derivatives have been developed and investigated for their therapeutic properties. For example, it has been revealed that 5,6-dihydroacridine derivatives possess antidiabetic and antioxidant properties.^25,26^ Therefore, it is highly important to prepare appropriate conditions for fast and direct synthesis of hydroacridine derivatives. Generally, HHAs and a wide spectrum of active pharmaceutical ingredients (APIs) are synthesized *via* multicomponent coupling reactions. Today, to obtain purer products with high reaction yields and to shorten the reaction time, many strategies are being introduced and applied. One of the most effective strategies is to use heterogeneous metallic catalytic systems.^27–29^ Briefly, through the existence of heteroatoms in the structure of the preliminary reactants of multicomponent reactions, constructive electronic interactions provide suitable conditions for chemical bonding. In this regard, sulfonated polymeric networks appear to be efficient for catalysis of organic synthesis reactions. For instance, a polymer-impregnated sulfonated carbon composite was recently reported as an acidic catalytic system for assisting the alkylation of phenol.^30^ In this study, our aim was to sulfonate acacia gum and apply it to facilitate the multicomponent synthesis reactions of tetramethyl-9-phenyl-hexahydroacridine-1,8(2*H*,5*H*)-dione. The general structure of these pharmaceuticals is presented in Fig. 1(b).

Concisely, we introduce a convenient method to synthesize an Fe_3_O_4_@acacia–SO_3_H heterogeneous magnetic catalytic system. Then, it is clearly shown that the synthesis of HHA derivatives is highly facilitated through applying this efficient catalyst. 87–94% reaction yields were obtained for different derivatives of HHA in reaction times of less than two hours. Moreover, convenient separation and excellent reusability were observed for this system through its magnetic properties.

## 2. Results and discussion

### 2.1. Preparation method of the Fe_3_O_4_@acacia–SO_3_H nano-powder

**Fig. 2:**
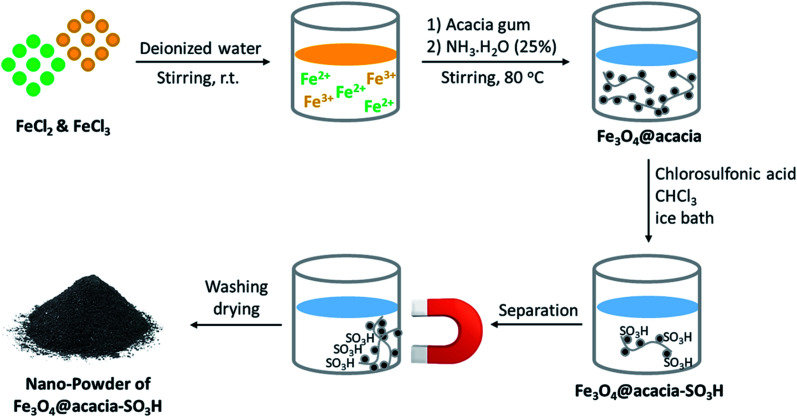
Schematic of the preparation route of the Fe_3_O_4_@acacia–SO_3_H nano-powder.

As presented in Fig. 2, iron(ii) and iron(iii) chloride salts were dissolved in deionized water at room temperature. Then, acacia gum powder was added and was also dissolved. In the next stage, iron ions were precipitated *via* co-deposition and produced Fe_3_O_4_ nanoparticles, which were well composed with the polymeric texture of acacia.^31^*Via* this *in situ* method, a better composition was obtained, and the dark particles of Fe_3_O_4_ were well immobilized. For this purpose, ammonia solution was used to raise the pH value. Moreover, after separation and drying of the precipitate, the particles of Fe_3_O_4_@acacia were dispersed in chloroform and the temperature was reduced by an ice bath. Due to the exothermic reaction of sulfonic acid, gentle addition of this material at cool temperatures is required. During the preparation process, the Fe_3_O_4_ nanoparticles appear to electrostatically combine with the acacia textures because both species contain several hydroxyl groups in their chemical structures. In the case of sulfone groups, they are most likely covalently attached to the acacia and Fe_3_O_4_ nanoparticles.^32^ In the next stage, after completion of the addition of sulfonic acid and stirring for 120 min, the particles of Fe_3_O_4_@acacia–SO_3_H composite were magnetically separated, washed, and dried in an oven.

### 2.2. Characterization of the Fe_3_O_4_@acacia–SO_3_H nano-powder

#### 2.2.1. FT-IR and EDX studies

**Fig. 3:**
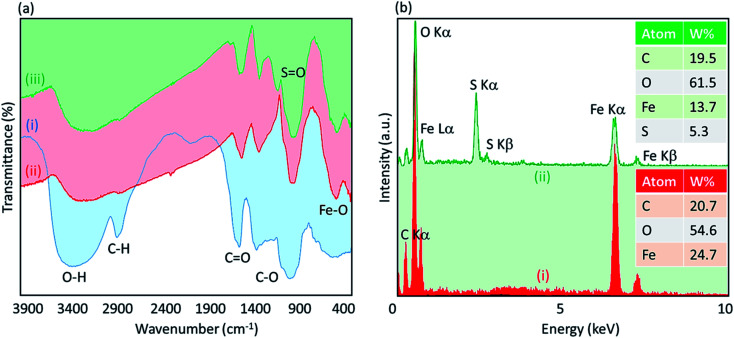
(a) Fourier-transform infrared spectra of (i) the neat acacia gum, (ii) Fe_3_O_4_@acacia binary composite, and (iii) Fe_3_O_4_@acacia–SO_3_H nano-powder; (b) energy-dispersive X-ray spectra of (i) the Fe_3_O_4_@acacia binary composite and (ii) the fabricated Fe_3_O_4_@acacia–SO_3_H nano-powder.

To investigate the presence of essential functional groups in the structure of the Fe_3_O_4_@acacia–SO_3_H nano-powder, Fourier-transform infrared (FT-IR) spectra of the neat acacia gum (spectrum i), Fe_3_O_4_@acacia binary composite (spectrum ii), and Fe_3_O_4_@acacia–SO_3_H nano-powder (spectrum iii) were acquired and are presented in Fig. 3(a). As can be seen in the spectra, the presence of the O–H, C–H (hybridation sp^3^), and C–O bands was confirmed by the peaks appearing at 3400, 2929, 1050 and 1250 cm^−1^, respectively. Also, it appears that some of the hydroxyl groups in the structure of the acacia gum were converted to C=O. This claim is proven by the peak that appeared at ∼1660 cm^−1^ in the spectrum (i). The composition of the Fe_3_O_4_ NPs was confirmed by the peak appearing at ∼590 cm^−1^ in the spectrum (ii), which is related to the Fe–O bond. As observed in the spectrum (ii), the sharp broad peak of hydroxyl groups in the structure of acacia gum became deformed; this result may be due to the physicochemical composition of the Fe_3_O_4_ NPs. According to literature, the peak related to the S=O bond is appeared in the range of 1000–1200 cm^−1^. Accordingly, as shown in the spectrum (iii), this peak appeared and confirmed the successful sulfonation of the Fe_3_O_4_@acacia binary composite. To obtain more confirmation of the successful execution of the sulfonation process, energy-dispersive X-ray (EDX) analysis was also performed. As Fig. 3(b) shows, 5.3% of the total weight of the Fe_3_O_4_@acacia–SO_3_H nanocomposite was formed of sulfur after carrying out the sulfonation process. The existence of the other essential elements, such as carbon, oxygen, and iron, related to the desired structures of the Fe_3_O_4_@acacia binary composite and Fe_3_O_4_@acacia–SO_3_H nano-powder are also proven by EDX analysis.

#### 2.2.2. TGA and VSM studies

**Fig. 4:**
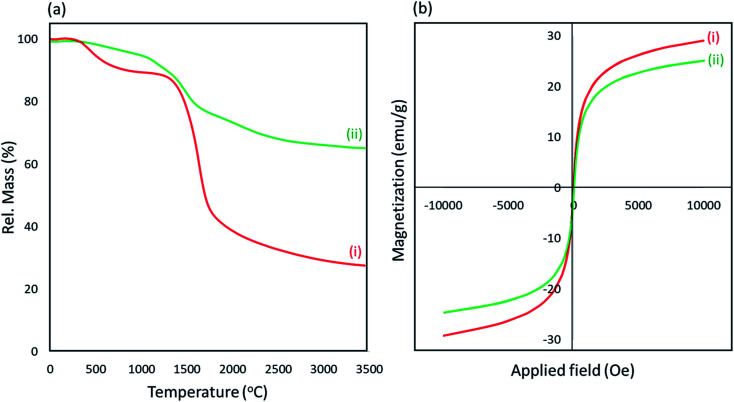
(a) Thermogravimetric analysis curves and (b) room-temperature *M*–*H* curves of the (i) Fe_3_O_4_@acacia binary composite and (ii) fabricated Fe_3_O_4_@acacia–SO_3_H nano-powder.

To check thermal stability of our prepared Fe_3_O_4_@acacia–SO_3_H nano-powder, thermogravimetric analysis (TGA) was performed in a thermal range of 0–3500 °C (Fig. 4(a)). This method also gives some information about the combination of the Fe_3_O_4_ NPs and the sulfonated acacia *via* monitoring of the decomposition process. For the Fe_3_O_4_@acacia binary composite (curve i), it can be clearly observed that proportional to the temperature rise, two distinct shoulders in the thermal ranges of 0–700 °C and 800–1700 °C appeared; then, the weight percentage gradually decreased from ∼1700 °C onwards. The first shoulder can be related to the dehydroxylation process of the acacia gum. Reportedly, the organic layers and the hydroxyl groups are separated from the structure as hydrate molecules up to 700 °C.^33^ In the next stage, in which ∼55% of the total weight was lost, the acacia gum likely decomposed and the individual Fe_3_O_4_ NPs started to collapse from ∼1700 °C. In curve (ii), which belongs to the fabricated Fe_3_O_4_@acacia–SO_3_H nano-powder, it can be clearly observed that the stability of the organic functional groups was significantly enhanced and the dehydroxylation process was prolonged to ∼1500 °C instead of 700 °C. Then, the decomposition process started from ∼1600 °C, and the weight was gradually reduced. It can also be seen that only 40% of the total weight was lost up to 3500 °C; this indicates that the general stability of the fabricated nano-powder was enhanced *via* the composition process. The magnetic property of the desired product was also studied by vibrating-sample magnetometry (VSM), and a comparison was made with the Fe_3_O_4_@acacia binary composite through their magnetic-hysteresis (*M*–*H*) curves (Fig. 4(b)). As shown, the magnetic property of the Fe_3_O_4_@acacia binary composite (curve i) decreased slightly (∼4.0 emu g^−1^) after performing the sulfonation process. The most probable reason is removal of some of the Fe_3_O_4_ magnetic NPs that were not strongly attached to the polymeric fibers during the sulfonation process. However, magnetic saturation for the fabricated Fe_3_O_4_@acacia–SO_3_H nano-powder occurred at ∼23.5 emu g^−1^ by applying a magnetic field with 10 000 (Oe) power, and this value is enough to perform a convenient magnetic separation process.

#### 2.2.3. XRD study

**Fig. 5:**
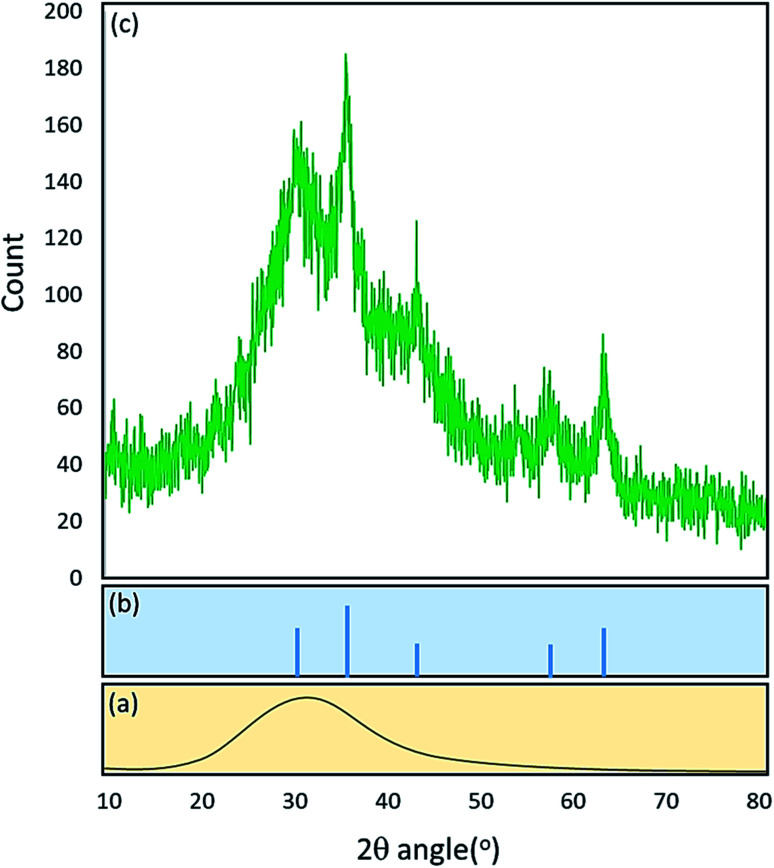
X-ray diffraction patterns of (a) neat acacia gum, (b) the Fe_3_O_4_ NPs, and (c) the fabricated Fe_3_O_4_@acacia–SO_3_H nano-powder.

The X-ray diffraction (XRD) pattern of the prepared Fe_3_O_4_@acacia–SO_3_H nano-powder was also investigated to check the effects of the composited ingredients on the general crystal structure (Fig. 5). With a quick look at the spectrum, the presence of a broad peak starting from 2*θ* = 20° and continuing to 40° is confirmed. According to the literature, this broad peak is related to the crystal structure of neat acacia gum.^34^ This result indicates that the acacia polymeric network does not include a well-defined crystal structure in comparison with the inorganic components. Also, there are some other peaks in the XRD spectrum that are relatively sharp and can be considered as indicative signals of the Fe_3_O_4_ inorganic crystal structure. *Via* a comparison with the reference pattern of the Fe_3_O_4_ NPs (JCPDS #99-0073), it was revealed that the peaks appearing at 2*θ* = 30.7, 36.2, 43.4, 57.7, and 63.4° belong to the crystal structure of the composited Fe_3_O_4_ NPs. These peaks are also associated with the Miller indices (2 2 0), (3 1 1), (4 0 0), (5 1 1), and (4 4 0), respectively.

#### 2.2.4. EM study

**Fig. 6:**
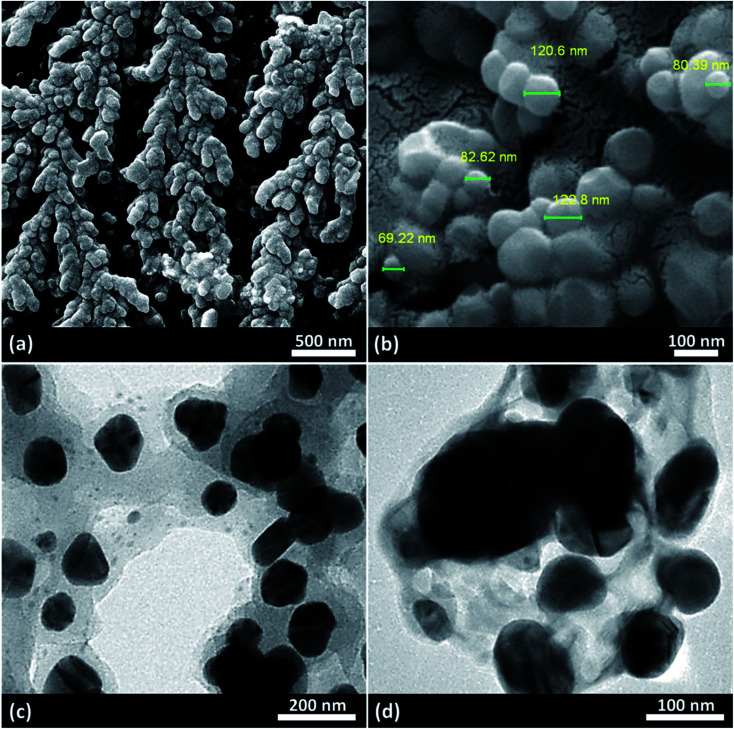
(a and b) Field-emission scanning electron microscopy and (c and d) transmission electron microscopy images of the fabricated Fe_3_O_4_@acacia–SO_3_H nano-powder.

One of the most preferred methods for investigating the sizes, morphologies, and compositions of microscale and nanoscale materials is electron microscopy (EM). This is because EM gives direct information from the samples without any need for further interpretation or inaccurate estimations. Fig. 6 illustrates the field-emission scanning electron microscopy (FESEM) (images a and b) and transmission electron microscopy (TEM) images (images c and d) of the fabricated Fe_3_O_4_@acacia–SO_3_H nano-powder at different magnifications. As can be observed in all the images, the mean size of the captured Fe_3_O_4_ NPs between the acacia textures is around 86 nm. Also, high uniformity in the sizes and shapes of the particles as well as a monotonous distribution onto the acacia gum fibers are nicely illustrated in image (a). Obviously, this good dispersion of the particles provides an extremely active surface area for catalytic applications. The TEM images also clearly disclosed that the spherical-shaped NPs were entrapped in the polymeric matrix. This composition may lead to higher mechanical stability in catalytic systems. This stability will be better highlighted in the recycling process investigation.

### 2.3. Catalytic application of the Fe_3_O_4_@acacia–SO_3_H nano-powder in the organic synthesis of 9-phenyl hexahydroacridine pharmaceutical derivatives

**Scheme 1:**
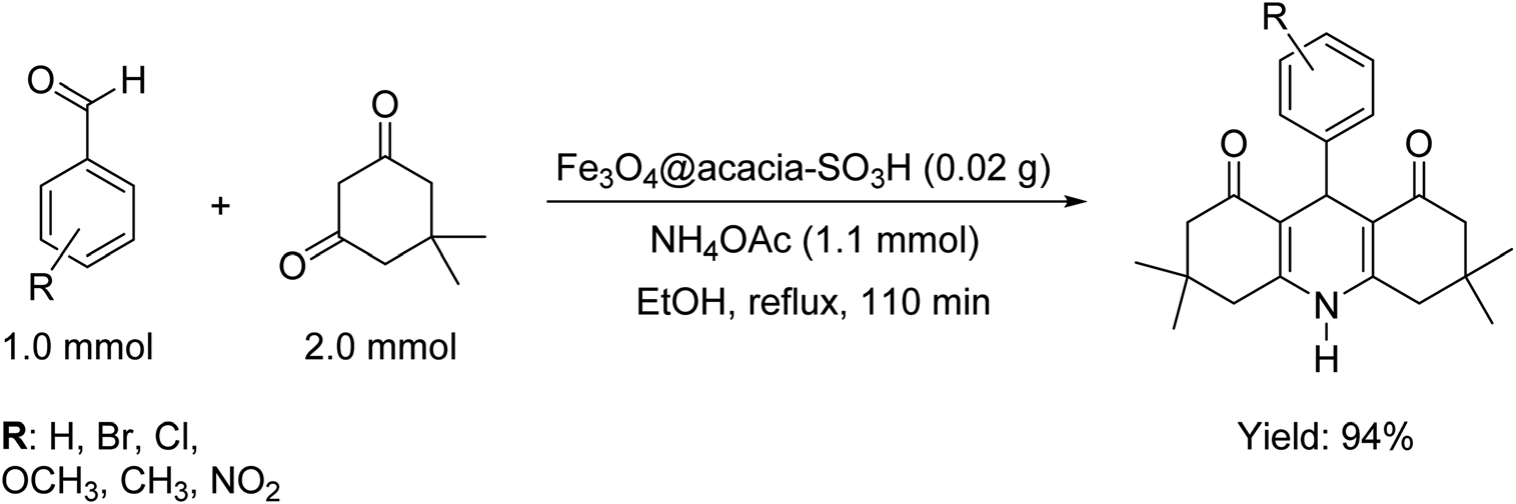
General schematic of the organic synthesis reaction of the 9-phenyl hexahydroacridine derivatives catalyzed by the Fe_3_O_4_@acacia–SO_3_H nanocatalyst.

As discussed in the Introduction section, the main goal of the design and fabrication of the Fe_3_O_4_@acacia–SO_3_H nano-powder was to provide a suitably active substrate with high heterogeneity to increase the convenience of the organic synthesis of 9-phenyl hexahydroacridine pharmaceutical derivatives. Here, it is clearly demonstrated that high reaction yields were obtained through applying the present catalytic system. Moreover, the reaction time significantly decreased in comparison with the catalyst-free conditions. A brief comparison was made between our novel designed catalytic system and other recently reported systems that highlights the high efficiency of the present nanocomposite in organic catalysis (Table 1). Scheme 1 presents a general view of the targeted organic reaction that was intended to be catalyzed by the Fe_3_O_4_@acacia–SO_3_H nano-powder.

**Table 1:** Optimization information for the catalyzed synthesis reaction of 9-(4-methoxyphenyl)-3,3,6,6-tetramethyl-3,4,6,7,9,10-hexahydroacridine-1,8(2*H*,5*H*)-dione^a^

Entry	Cat. system	Cat. weight (g)	Medium	Temp. (°C)	Time (min)	Yield^b^ (%)
1	—	—	EtOH	25	110	N.R.
2	—	—	EtOH	75	110	N.R.
3	Fe_3_O_4_ NPs	0.02	EtOH	75	110	Trace
4	Acacia gum	0.02	EtOH	75	110	Trace
5	Fe_3_O_4_@acacia–SO_3_H	0.01	EtOH	75	110	88
6	Fe_3_O_4_@acacia–SO_3_H	0.02	EtOH	75	110	94^c^
7	Fe_3_O_4_@acacia–SO_3_H	0.02	EtOH	75	300	94
8	Fe_3_O_4_@acacia–SO_3_H	0.03	EtOH	75	110	94
9	Fe_3_O_4_@acacia–SO_3_H	0.03	EtOH	50	110	91
10	Fe_3_O_4_@acacia–SO_3_H	0.02	H_2_O	80	110	62
11	Fe_3_O_4_@acacia–SO_3_H	0.02	DMF	130	110	75
12	Fe_3_O_4_@acacia–SO_3_H	0.02	DCM	35	110	79
13	Fe_3_O_4_@acacia–SO_3_H	0.02	Toluene	130	110	76
14	Fe_3_O_4_@acacia–SO_3_H	0.02	CH_3_CN	75	110	79
15	Nano-Fe_3_O_4_–TiO_2_–SO_3_H	0.01	Solvent free	110	55	86 (ref. 35)
16	Fe_3_O_4_@SiO_2_–MoO_3_H	0.02	Solvent free	90	40	90 (ref. 36)
17	Cell-Pr-NHSO_3_H	0.05	Ethanol	Reflux	48	88 (ref. 37)

a Abbreviations: Cat.: catalyst; Temp.: temperature, DMF: dimethylformamide; DCM: dichloromethane; N.R.: no reaction. The reaction progress was controlled by thin-layer chromatography, and the desired hexahydroacridine product was purified *via* flash-column chromatography.

b Isolated yield.

c Optimum conditions.

#### 2.3.1. Optimization

Concisely, various conditions, including catalyst-free reactions, reactions catalyzed by the neat Fe_3_O_4_ NPs and acacia gum powder individually, and catalytic systems with different amounts of Fe_3_O_4_@acacia–SO_3_H, various reaction media and different reaction times were precisely monitored in the synthesis reaction of 9-(4-methoxyphenyl)-3,3,6,6-tetramethyl-3,4,6,7,9,10-hexahydroacridine-1,8(2*H*,5*H*)-dione, which was considered as a model reaction. Table 1 briefly reports the obtained results from each case and also shows that 94% yield was obtained through using 0.02 g of Fe_3_O_4_@acacia–SO_3_H nanocomposite under reflux conditions.

#### 2.3.2. Synthesis of 9-phenyl hexahydroacridines catalyzed by Fe_3_O_4_@acacia–SO_3_H nano-powder

To investigate the catalytic performance of the fabricated Fe_3_O_4_@acacia–SO_3_H nanocatalyst, various derivatives of the aldehyde component, including bromine, chlorine, methyl, methoxy, and nitro groups, were studied under the optimal conditions. For initial assessment of the desired products, melting point measurements were used. Then, some of the products were selected and identified *via* spectroscopic methods. Table 2 reports the synthesized products *via* the presented catalytic process.

**Table 2:** Various derivatives of 9-phenyl hexahydroacridine synthesized *via* the catalytic process using the Fe_3_O_4_@acacia–SO_3_H nanocatalyst

Entry	Product structure	Product code	Time (min)	Yield^a^ (%)	Melting point (°C)		Ref.
					Found	Reported	
1	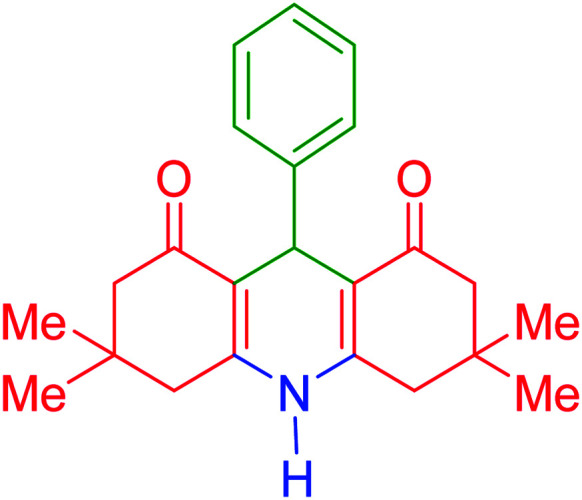	a	110	93	279–281	277–279	38
2	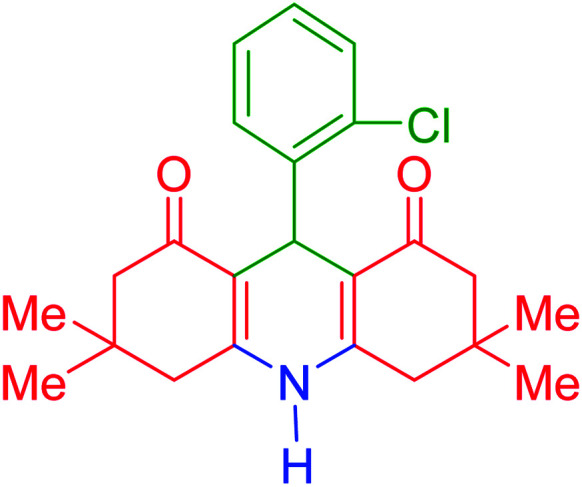	b	145	87	264–266	263–264	39
3	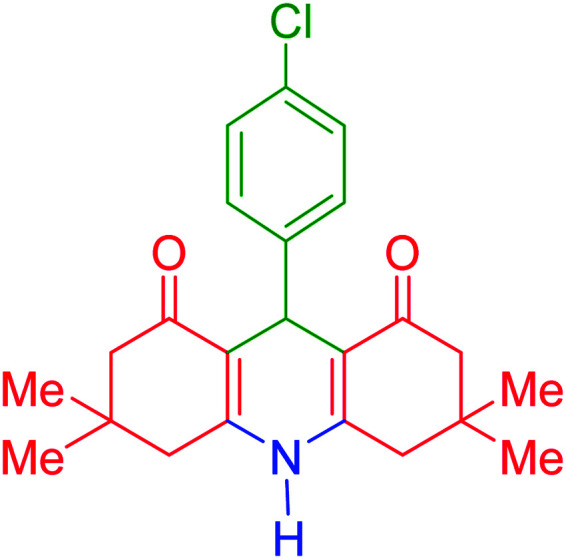	c	135	91	290–292	295–297	40
4	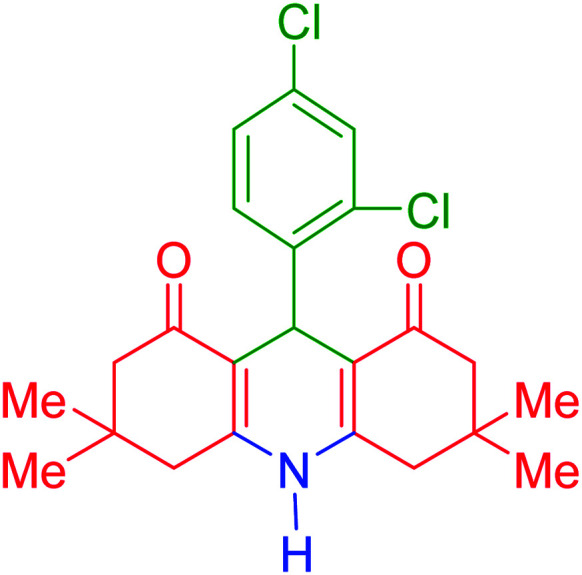	d	150	87	318–320	319–321	41
5	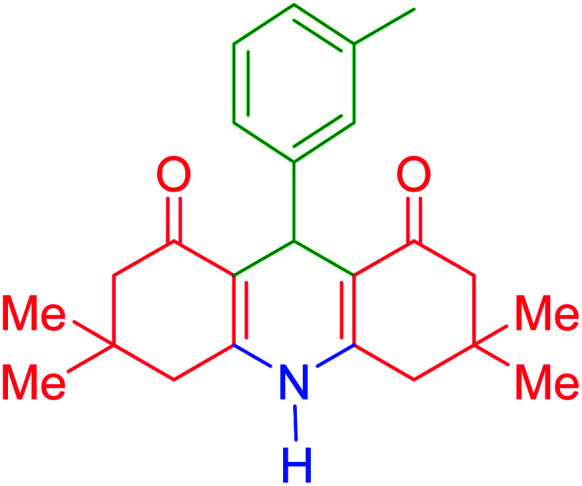	e	125	91	211–213	210–213	41
6	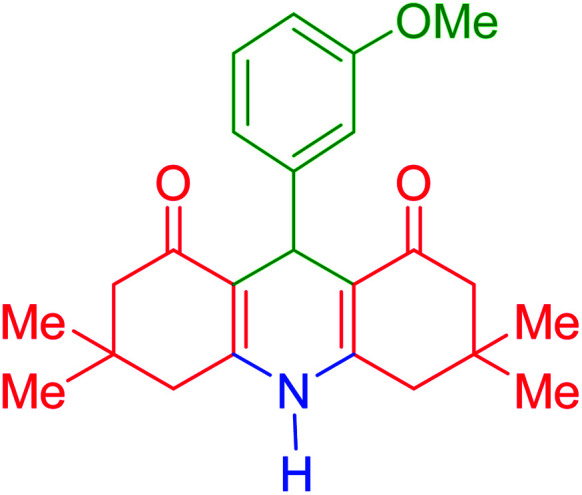	f	120	92	301–303	300–302	42
7	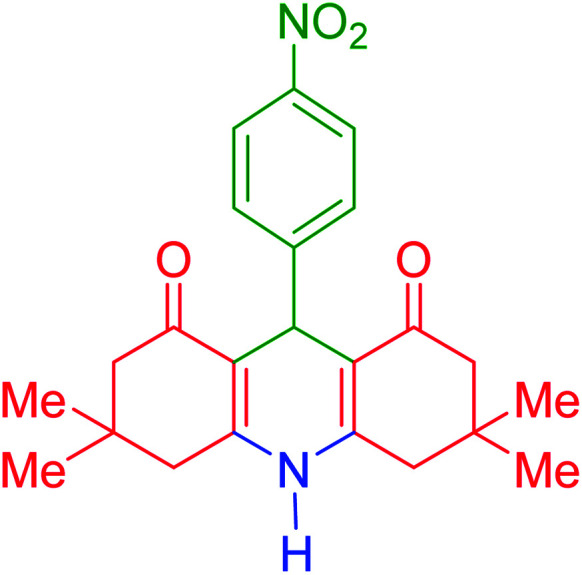	g	150	86	286–288	287–289	36
8	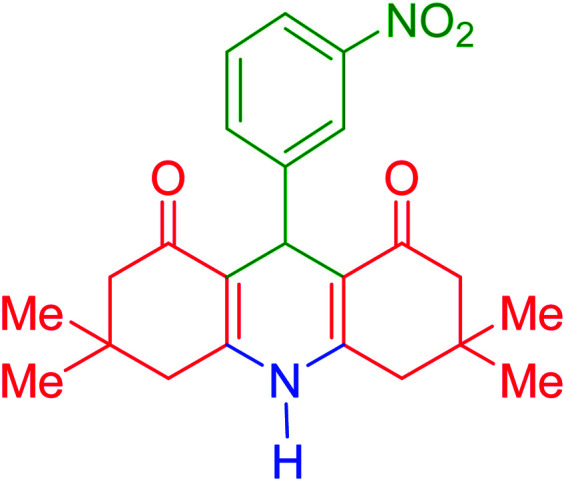	h	150	86	281–283	282–284	43
9	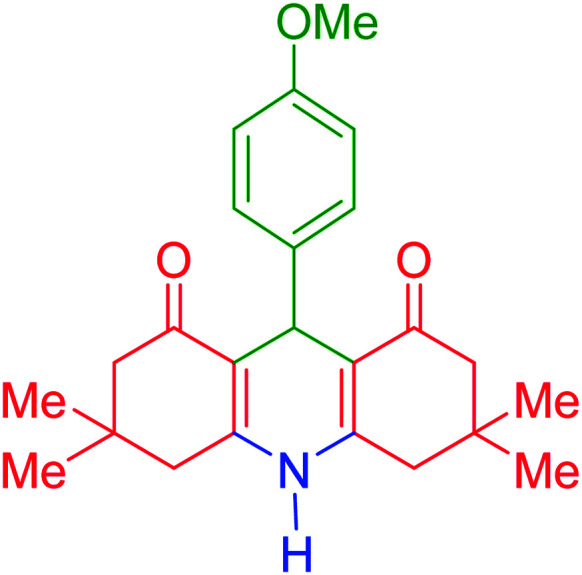	i	110	94	288–290	287–290	44
10	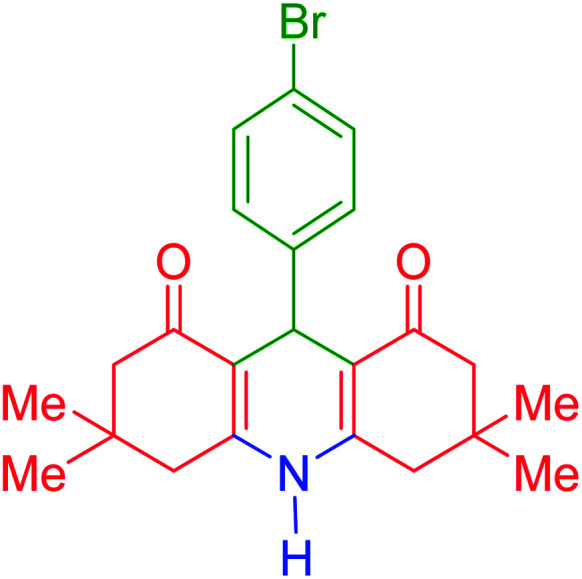	j	140	90	321–324	322–324	45

a Isolated yield.

#### 2.3.3. Suggested mechanism of the catalytic activity of the Fe_3_O_4_@acacia–SO_3_H nano-powder

**Scheme 2:**
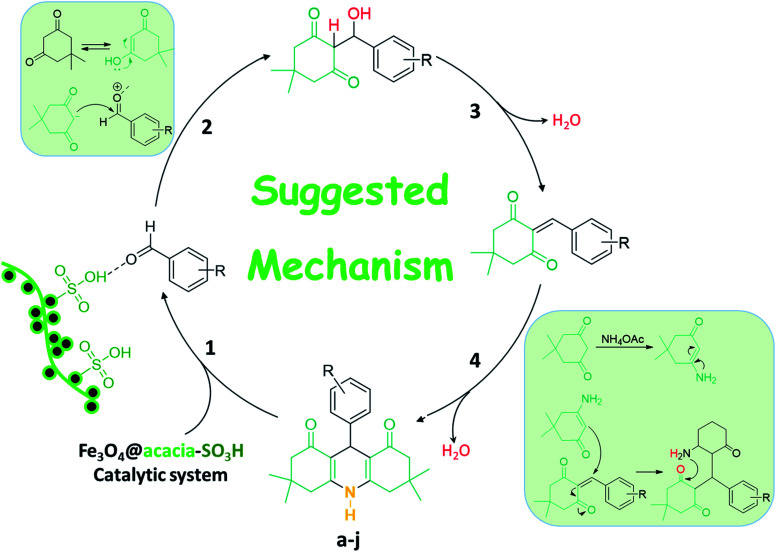
Plausible mechanism of the catalytic activity of the fabricated Fe_3_O_4_@acacia–SO_3_H nanocatalyst in the synthesis reactions of 9-phenyl hexahydroacridine derivatives.

The Fe_3_O_4_@acacia–SO_3_H nano-powder is an acidic catalytic system in which the catalysis proceeds *via* H-bonding interactions with the involved ingredients in the synthesis reactions. As a plausible mechanism, Fe_3_O_4_@acacia–SO_3_H starts with activation of the aldehyde component in the first stage. Dimedone enters the cycle by performing a nucleophilic attack on the activated aldehyde (stage 2). Next, a π-conjugated system is formed during a dehydration process (stage 3). Afterward, in stage four, another dimedone performs a nucleophilic attack on the structure of the conjugated compound; then, NH_4_ enters the cycle and forms the structure of the target 9-phenyl hexahydroacridine. Scheme 2 schematically presents the explained catalytic cycle.^46–51^

#### 2.3.4. Recyclability of the Fe_3_O_4_@acacia–SO_3_H catalytic system

**Fig. 7:**
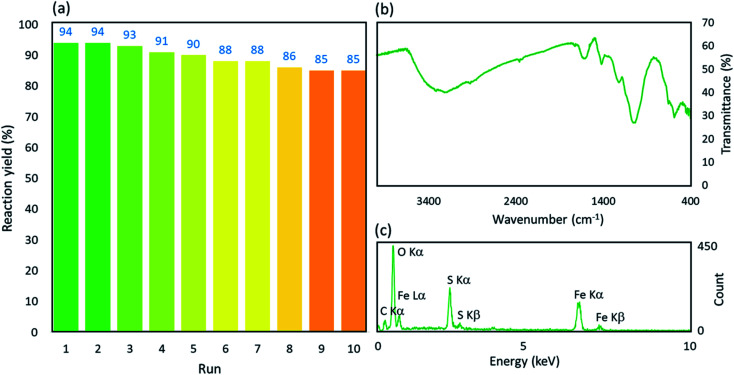
(a) Recycling diagram, (b) Fourier-transform infrared spectrum, and (c) energy-dispersive X-ray spectrum of the recovered Fe_3_O_4_@acacia–SO_3_H catalytic system.

The stability of the fabricated catalytic system was precisely investigated by successive running of the catalytic process in the synthesis reaction of product i. As can be seen in Fig. 7(a), acceptable reaction yields were obtained in a total of ten runs of the reaction. After recycling and reusing the nanoparticles ten times, FT-IR and EDX spectra of the recovered nanocomposite were prepared and investigated. From these analyses, it was clearly revealed that no significant changes occurred in the structure of the Fe_3_O_4_@acacia–SO_3_H catalytic system. As can be observed in Fig. 7(b and c), all of the distinct indicative peaks appeared in both spectra. Moreover, inductively coupled plasma (ICP) analysis was performed to investigate the metal leaching from the system. After completion of the catalytic process (after run 1), the particles were separated and the supernatant was filtered and analyzed. Briefly, it was observed that only 0.15 mg of the iron element leached from 0.05 g of the catalytic system.

## 3. Experimental

### 3.1. Materials and equipment

All commercially available chemicals, solvents, reagents and were purchased from Sigma-Aldrich and Merck Company. All the applied materials and equipment are summarized in Table S1.†

### 3.2. Practical methods

#### 3.2.1. Preparation of Fe_3_O_4_@acacia binary composite

In a round bottom flask (50 mL), FeCl_2_·4H_2_O and FeCl_3_·6H_2_O salts (1.0 mmol and 2.0 mmol, respectively) were dissolved in deionized water (10 mL) *via* vigorous stirring at room temperature. Then, acacia gum powder (0.6 g) was added in several portions and also dissolved. In the next stage, the reaction mixture was gradually heated to around 80 °C under a neutral atmosphere of N_2_. Then, ammonia solution (13 mL) was added dropwise until the pH value reached ∼12. The dark mixture was then stirred under the same conditions for an additional 1 h. Finally, the magnetic particles were collected *via* holding an external magnet at the bottom of the flask after cooling to room temperature. The particles were washed with ethanol and water several times and dried in an oven at 60 °C.

#### 3.2.2. Preparation of Fe_3_O_4_@acacia–SO_3_H nano-powder

In a round bottom flask (50 mL), the particles of Fe_3_O_4_@acacia (0.6 g) were dispersed in chloroform (10 mL), and the temperature was reduced by an ice bath. In a separate flask, chlorosulfonic acid (99%) (2.0 mL) was mixed with chloroform (2.0 mL), and the resulting solution was added dropwise to the main reaction flask with stirring. After completion of the addition, the ice bath was removed, and vigorous stirring was continued for an additional 2 h at room temperature. Ultimately, the product was magnetically separated, washed, and dried as described above.

#### 3.2.3. General procedure for the catalyzed synthesis of 9-phenyl hexahydroacridine pharmaceutical derivatives

In a round bottom flask (25 mL), aldehyde (1.0 mmol), dimedone (2.0 mmol), ammonium acetate salt (1.1 mmol), and Fe_3_O_4_@acacia–SO_3_H nano-powder (0.02 g) were mixed in ethanol (2.0 mL), and the mixture was refluxed. After the appropriate time had passed (110 min), the particles of the catalytic system were magnetically removed and the desired product was purified *via* flash-column chromatography. The original ^1^H and ^13^C-NMR spectra of the selected products are shown in Fig. S1–S20 in the ESI† section.

#### 3.2.4. Recycling of the catalyst

After completion of the first round, the Fe_3_O_4_@acacia–SO_3_H particles were magnetically separated and the rest were separated *via* decanting. Then, the particles were washed well with deionized water and ethanol (20 mL) four successive times. Afterward, the particles were died in a vacuum oven for 24 h. To reuse the particles, redispersion was initially performed by an ultrasound cleaner bath (50 kHz, 200 W L^−1^); then, the reactants were added to the flask.

### 3.3. Spectral data for selected products

3,3,6,6-Tetramethyl-9-phenyl-3,4,6,7,9,10-hexahydroacridine-1,8(2*H*,5*H*)-dione (product a): M. P (°C): 279–281. ^1^H NMR (300 MHz, DMSO), *δ* (ppm): 9.30 (s, 1H), 7.00–7.13 (m, 5H), 4.60 (s, 1H), 2.40–2.48 (m, 2H), 2.27 (d, 2H), 2.12 (d, 2H), 2.00 (d, 2H), 0.98 (s, 6H), 0.83 (s, 6H). ^13^C NMR (75 MHz, DMSO), *δ* (ppm): 194.3, 149.3, 147.1, 127.6, 127.5, 125.4, 111.4, 50.2, 32.9, 32.2, 29.1, 26.4.

9-(2-Chlorophenyl)-3,3,6,6-tetramethyl-3,4,6,7,9,10-hexahydroacridine-1,8(2*H*,5*H*)-dione (product b): M. P (°C): 264–266. ^1^H NMR (300 MHz, DMSO), *δ* (ppm): 9.59 (s, 1H), 7.25–7.27 (d, 1H), 7.10–7.20 (m, 1H), 7.04–7.08 (m, 1H), 6.99–7.01 (m, 1H), 5.05 (s, 1H), 2.71–2.10 (m, 8H), 0.98 (s, 6H), 0.92 (s, 6H). ^13^C NMR (75 MHz, DMSO), *δ* (ppm): 196.4, 152.4, 142.6, 132.0, 129.7, 128.2, 115.2, 50.6, 40.8, 32.2, 31.4, 29.3, 27.2.

9-(4-Chlorophenyl)-3,3,6,6-tetramethyl-3,4,6,7,9,10-hexahydroacridine-1,8(2*H*,5*H*)-dione (product c): M. P (°C): 290–292. ^1^H NMR (300 MHz, DMSO), *δ* (ppm): 9.92 (s, 1H), 7.27–7.30 (d, 2H), 7.18–7.19 (d, 2H), 4.50 (s, 1H), 2.50–2.59 (dd, 4H), 2.25–2.28 (d, 2H), 2.07–2.10 (d, 2H), 1.04 (s, 6H), 0.90 (s, 6H). ^13^C NMR (75 MHz, DMSO), *δ* (ppm): 196.1, 149.3, 147.1, 131.7, 129.6, 129.3, 128.2, 113.1, 50.2, 33.6, 32.8, 29.1, 27.3.

9-(2,4-Dichlorophenyl)-3,3,6,6-tetramethyl-3,4,6,7,9,10-hexahydroacridine-1,8(2*H*,5*H*)-dione (product d): M. P (°C): 318–320. ^1^H NMR (300 MHz, DMSO), *δ* (ppm): 9.95 (s, 1H), 7.34 (s, 1H), 7.28–7.33 (d, 1H), 7.19–7.22 (d, 1H), 5.58 (s, 1H), 2.38–2.43 (m, 2H), 2.34 (d, 2H), 2.32 (d, 2H), 2.31 (d, 2H), 1.14 (s, 6H), 1.07 (s, 6H). ^13^C NMR (75 MHz, DMSO), *δ* (ppm): 189.8, 135.3, 134.1, 130.0, 129.5, 126.7, 115.4, 47.0, 46.9, 46.3, 31.7, 31.2, 28.9, 27.8.

9-(3-Methylphenyl)-3,3,6,6-tetramethyl-3,4,6,7,9,10-hexahydroacridine-1,8(2*H*,5*H*)-dione (product e): M. P (°C): 211–213. ^1^H NMR (300 MHz, DMSO), *δ* (ppm): 8.07 (s, 1H), 7.26 (s, 1H), 7.09–7.11 (d, 1H), 7.03–7.08 (m, 1H), 6.86–6.88 (d, 1H), 5.05 (s, 1H), 2.32–2.40 (m, 2H), 2.26 (d, 2H), 2.22 (d, 2H), 2.16 (d, 2H), 1.05 (s, 6H), 0.95 (s, 6H). ^13^C NMR (75 MHz, DMSO), *δ* (ppm): 196.1, 149.5, 146.7, 137.3, 129.1, 128.0, 126.9, 125.2, 113.3, 51.1, 40.8, 33.6, 32.7, 29.8, 27.2, 21.8.

9-(3-Methoxyphenyl)-3,3,6,6-tetramethyl-3,4,6,7,9,10-hexahydroacridine-1,8(2*H*,5*H*)-dione (product f): M. P (°C): 301–303. ^1^H NMR (300 MHz, DMSO), *δ* (ppm): 8.94 (s, 1H), 7.25 (s, 1H), 7.22 (d, 1H), 7.01 (m, 1H), 6.98 (d, 1H), 5.07 (s, 1H), 3.91 (s, 3H), 2.22–2.27 (m, 2H), 2.17 (d, 2H), 2.11 (d, 2H), 2.04 (d, 2H), 1.12 (s, 6H), 0.95 (s, 6H). ^13^C NMR (75 MHz, DMSO), *δ* (ppm): 196.1, 158.72, 149.9, 143.8, 134.9, 128.5, 127.7, 125.2, 112.8, 50.8, 40.2, 33.0, 32.3, 29.4, 27.9, 20.9.

9-(4-Nitrophenyl)-3,3,6,6-tetramethyl-3,4,6,7,9,10-hexahydroacridine-1,8(2*H*,5*H*)-dione (product g): M. P (°C): 286–288. 1H NMR (300 MHz, DMSO), *δ* (ppm): 9.90 (s, 1H), 7.19–7.21 (d, 2H), 7.12–7.14 (d, 2H), 4.76 (s, 1H), 2.42–2.49 (d, 4H), 2.14–2.32 (d, 2H), 1.95–1.98 (d, 2H), 0.99 (s, 6H), 0.84 (s, 6H). ^13^C NMR (75 MHz, DMSO), *δ* (ppm): 194.4, 149.5, 146.1, 129.9, 129.5, 127.5, 115.5, 111.1, 50.1, 40.0, 32.6, 32.2, 29.0, 26.4.

3,3,6,6-Tetramethyl-9-(3-nitrophenyl)-3,4,6,7,9,10-hexahydroacridine-1,8(2*H*,5*H*)-dione (product h): M. P (°C): 281–283. ^1^H NMR (300 MHz, DMSO), *δ* (ppm): 9.31 (s, 1H), 8.00–8.01 (d, 2H), 7.65–7.66 (d, 1H), 7.54–7.57 (t, 1H), 4.65 (s, 1H), 2.50–2.59 (dd, 4H), 2.27–2.30 (d, 2H), 2.08–2.12 (d, 2H), 1.04 (s, 6H), 0.91 (s, 6H). ^13^C NMR (75 MHz, DMSO), *δ* (ppm): 196.0, 149.4, 149.0, 131.1, 129.7, 122.3, 112.9, 51.0, 40.9, 33.9, 32.8, 29.7, 27.3, 21.1.

9-(4-Methoxyphenyl)-3,3,6,6-tetramethyl-3,4,6,7,9,10-hexahydroacridine-1,8(2*H*,5*H*)-dione (product i): M. P (°C): 288–290. ^1^H NMR (300 MHz, DMSO), *δ* (ppm): 9.20 (s, 1H), 7.05–7.07 (t, 2H), 6.76–6.80 (t, 2H), 4.46 (s, 1H), 3.70 (s, 3H), 2.56 (d, 2H), 2.49–2.51 (m, 2H), 2.27 (d, 2H), 2.09 (d, 2H), 1.03 (s, 6H), 0.91 (s, 6H). ^13^C NMR (75 MHz, DMSO), *δ* (ppm): 196.2, 157.5, 149.6, 139.2, 128.8, 125.2, 113.0, 50.8, 40.36, 32.6, 32.4, 29.5, 26.9.

9-(4-Bromophenyl)-3,3,6,6-tetramethyl-3,4,6,7,9,10-hexahydroacridine-1,8(2*H*,5*H*)-dione (product j): M. P (°C): 321–324. ^1^H NMR (300 MHz, DMSO), *δ* (ppm): 9.33 (s, 1H), 7.23–7.26 (dd, 2H), 6.87–6.92 (m, 2H), 4.73 (s, 1H), 2.26–2.46 (m, 4H), 2.19–2.22 (m, 2H), 2.15 (m, 4H), 1.10 (s, 6H), 0.99 (s, 6H). ^13^C NMR (75 MHz, DMSO), *δ* (ppm): 194.4, 149.6, 149.3, 147.1, 127.9, 127.8, 126.0, 111.9, 50.7, 32.2, 31.2, 29.2, 27.3.

## 4. Conclusions

In this work, we designed and fabricated a novel catalytic system with high heterogeneity and magnetic features to facilitate the MCR synthetic reactions of 9-phenyl hexahydroacridine pharmaceutical derivatives. A combination of acacia gum (gum arabic) with iron oxide magnetic particles on the nanoscale was used as a magnetized natural matrix. From the physicochemical aspect, through effective H-binding interactions, the organic and inorganic ingredients were firmly fixed and combined well with each other. EM imaging approaches indeed disclosed the composition of the composite. Then, the prepared Fe_3_O_4_@acacia binary composite was equipped with sulfone groups, which are considered to be the main active catalytic sites. Afterward, the high catalytic performance of the formed cluster-shaped composite was investigated in the organic synthesis reactions of 9-phenyl hexahydroacridine derivatives. The mechanical and thermal stability of the fabricated Fe_3_O_4_@acacia–SO_3_H nano-powder was also studied, and this substantial stability was highlighted in the recycling process. Overall, herein, we have made an effort to comprehensively study the structural features of the Fe_3_O_4_@acacia–SO_3_H nano-powder and demonstrate the catalytic performance of this product. Due to the high convenience of the synthesis process and the low prices of the used raw materials, this product is recommended for industrial applications.

## Conflicts of interest

The authors declare no conflict of interest.

## Supplementary Material

RA-010-D0RA07986C-s001
