# Expression of concern: Facile route to synthesize Fe_3_O_4_@acacia–SO_3_H nanocomposite as a heterogeneous magnetic system for catalytic applications

**DOI:** 10.1039/d4ra90043j

**Published:** 2024-04-23

**Authors:** Reza Taheri-Ledari, Mir Saeed Esmaeili, Zahra Varzi, Reza Eivazzadeh-Keihan, Ali Maleki, Ahmed Esmail Shalan

**Affiliations:** a Catalysts and Organic Synthesis Research Laboratory, Department of Chemistry, Iran University of Science and Technology (IUST) Tehran 16846-13114 Iran maleki@iust.ac.ir +98 21 73021584 +98 21 77240640-50; b Central Metallurgical Research and Development Institute (CMRDI) P. O. Box 87, Helwan Cairo 11421 Egypt a.shalan133@gmail.com; c BCMaterials, Basque Center for Materials, Applications and Nanostructures Martina Casiano, UPV/EHU Science Park, Barrio Sarriena s/n Leioa 48940 Spain

## Abstract

Expression of concern for ‘Facile route to synthesize Fe_3_O_4_@acacia–SO_3_H nanocomposite as a heterogeneous magnetic system for catalytic applications’ by Reza Taheri-Ledari *et al.*, *RSC Adv.*, 2020, **10**, 40055–40067, https://doi.org/10.1039/D0RA07986C.

The Royal Society of Chemistry is publishing this expression of concern in order to alert readers that concerns have been raised regarding the reliability of the FESEM and TEM images in Fig. 6a, b and d, as well as the EDX spectra in Fig. 3b and 7c. An investigation is underway, and an expression of concern will continue to be associated with the article until a final outcome is reached.

Laura Fisher

12th April 2024

Executive Editor, *RSC Advances*

## Supplementary Material

