# Supplementary material for: Facile route to synthesize Fe3O4@acacia–SO3H nanocomposite as a heterogeneous magnetic system for catalytic applications
Source: RSC Adv. 2020 Nov 4;10(66):40055–67. doi: 10.1039/d0ra07986c (PMC9057486; doi:10.1039/d0ra07986c)
Supplement: RA-010-D0RA07986C-s001 [file RA-010-D0RA07986C-s001.pdf]

Electronic Supplementary Material (ESI) for RSC Advances.
This journal is © The Royal Society of Chemistry 2020

Supporting Information

Facile Route to Synthesis Fe_3O_4@acacia-SO_3H Nanocomposite as Heterogeneous Magnetic System for Catalytic Applications

Reza Taheri-Ledari[1], Mir Saeed Esmaeili[1], Zahra Varzi[1], Reza Eivazzadeh-Keihan[1], Ali Maleki[1,] and Ahmed Esmail Shalan[2,3,]**

[1] Catalysts and Organic Synthesis Research Laboratory, Department of Chemistry, Iran University of Science and Technology (IUST), Tehran 16846-13114, Iran.

[2] Central Metallurgical Research and Development Institute (CMRDI), P.O. Box 87, Helwan, Cairo 11421, Egypt.

[3] BCMaterials, Basque Center for Materials, Applications and Nanostructures, Martina Casiano, UPV/EHU Science Park, Barrio Sarriena s/n, Leioa 48940, Spain.

**Corresponding authors. (A. M) Tel.: +98 21 77240640-50; fax: +98 21 73021584. E-mail address: maleki@iust.ac.ir; (A. E. S.) E-mail: a.shalan133@gmail.com*

Author's ORCID:

Reza Taheri-Ledari: https://orcid.org/0000-0002-6511-9411

Ali Maleki: https://orcid.org/0000-0001-5490-3350

Ahmed Esmail Shalan: https://orcid.org/0000-0002-3424-1609

**Content**	**Page**
**Table S1**. The applied materials and equipment	S3
**Figure S1**. ^{1}H-NMR spectrum of product ***a***	S4
**Figure S2**. ^{13}C-NMR spectrum of product ***a***	S5
**Figure S3**. ^{1}H-NMR spectrum of product ***b***	S6
**Figure S4**. ^{13}C-NMR spectrum of product ***b***	S7
**Figure S5**. ^{1}H-NMR spectrum of product ***c***	S8
**Figure S6**. ^{13}C-NMR spectrum of product ***c***	S9
**Figure S7**. ^{1}H-NMR spectrum of product ***d***	S10
**Figure S8**. ^{13}C-NMR spectrum of product ***d***	S11
**Figure S9**. ^{1}H-NMR spectrum of product ***e***	S12
**Figure S10**. ^{13}C-NMR spectrum of product ***e***	S13

**Figure S11**. ^{1}H-NMR spectrum of product ***f***	S14
**Figure S12**. ^{13}C-NMR spectrum of product ***f***	S15
**Figure S13**. ^{1}H-NMR spectrum of product ***g***	S16
**Figure S14**. ^{13}C-NMR spectrum of product ***g***	S17
**Figure S15**. ^{1}H-NMR spectrum of product ***h***	S18
**Figure S16**. ^{13}C-NMR spectrum of product ***h***	S19
**Figure S17**. ^{1}H-NMR spectrum of product ***i***	S20
**Figure S18**. ^{13}C-NMR spectrum of product ***i***	S21
**Figure S19**. ^{1}H-NMR spectrum of product ***j***	S22
**Figure S20**. ^{13}C-NMR spectrum of product ***j***	S23

Table S1. The applied materials and equipment.

Material / equipment	Purity / Brand
$FeCl_2.4H_2O$	Sigma Aldrich, 98%
$FeCl_3.6H_2O$	Sigma Aldrich, 98%
Ammonium acetate	Merck
Acacia gum	Sigma Aldrich
Solvents	Merck
Aldehyde derivatives	Sigma Aldrich
Dimedone	Sigma Aldrich, 95%
Chlorosulfonic acid	Sigma Aldrich, 99%
Silica gel for column chromatography	Sigma Aldrich, 60%
Ultrasound bath	KQ-250 DE - 50 kHz and power of 250 W L^{-1}
Melting point measurement apparatus	Electrothermal 9100
FT-IR spectroscopy	Shimadzu IR-470
EDX spectroscopy	Numerix DXP-X10P
TGA apparatus	Bahr-STA 504 instrument under argon atmosphere
VSM apparatus	Lakeshore 7407 and Meghnatis Kavir Kashan Co., Iran
XRD spectroscopy	DRON-8 X-ray diffractometer
FESEM analysis	Sigma-Zeiss microscope with attached camera
TEM analysis	Philips CM200
NMR spectroscopy	Bruker DRX-300 Avance spectrometer at 300MHz
ICP	Agilent 7500ce

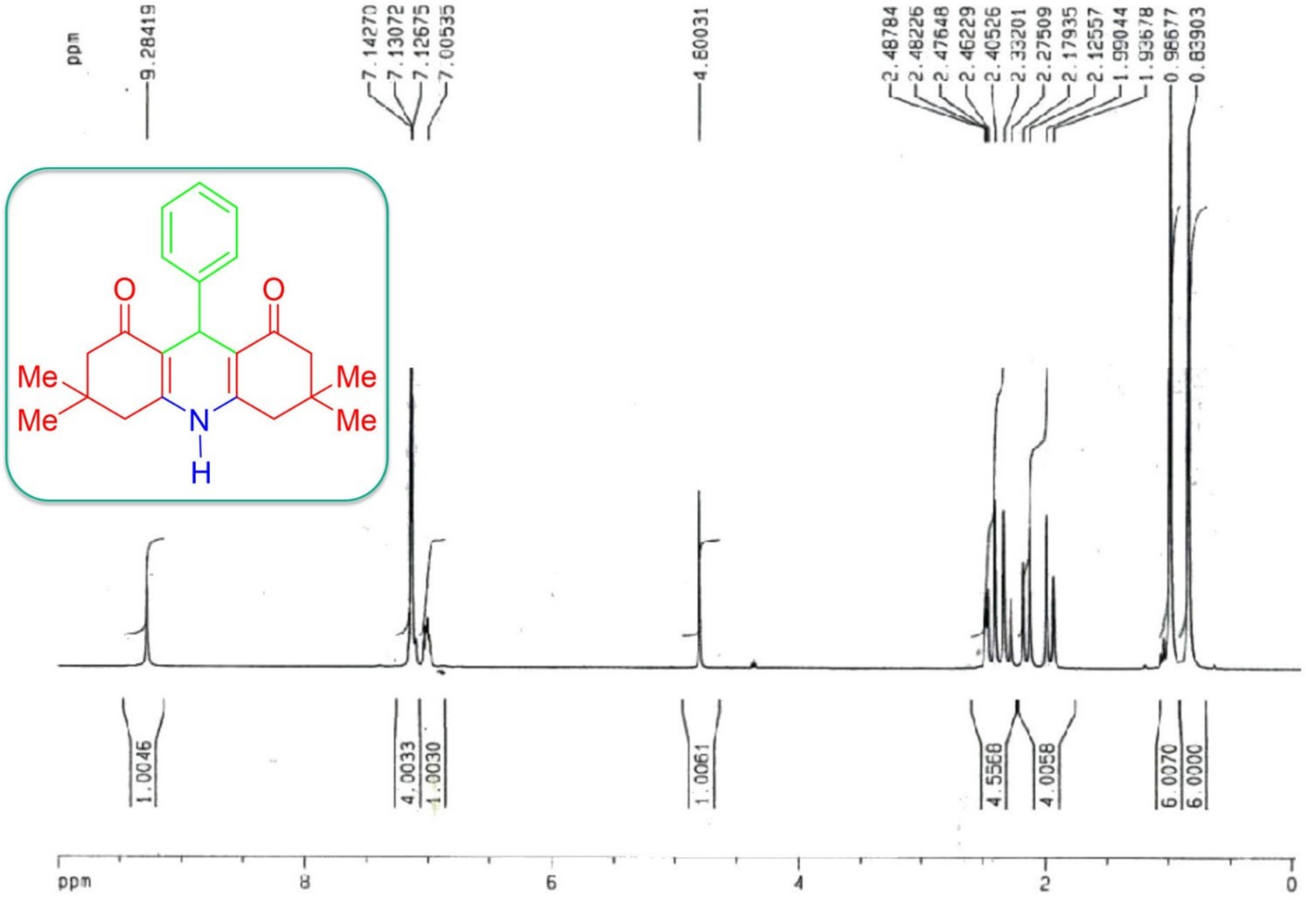

Figure S1. ^{1}H-NMR spectrum of product ***a.***

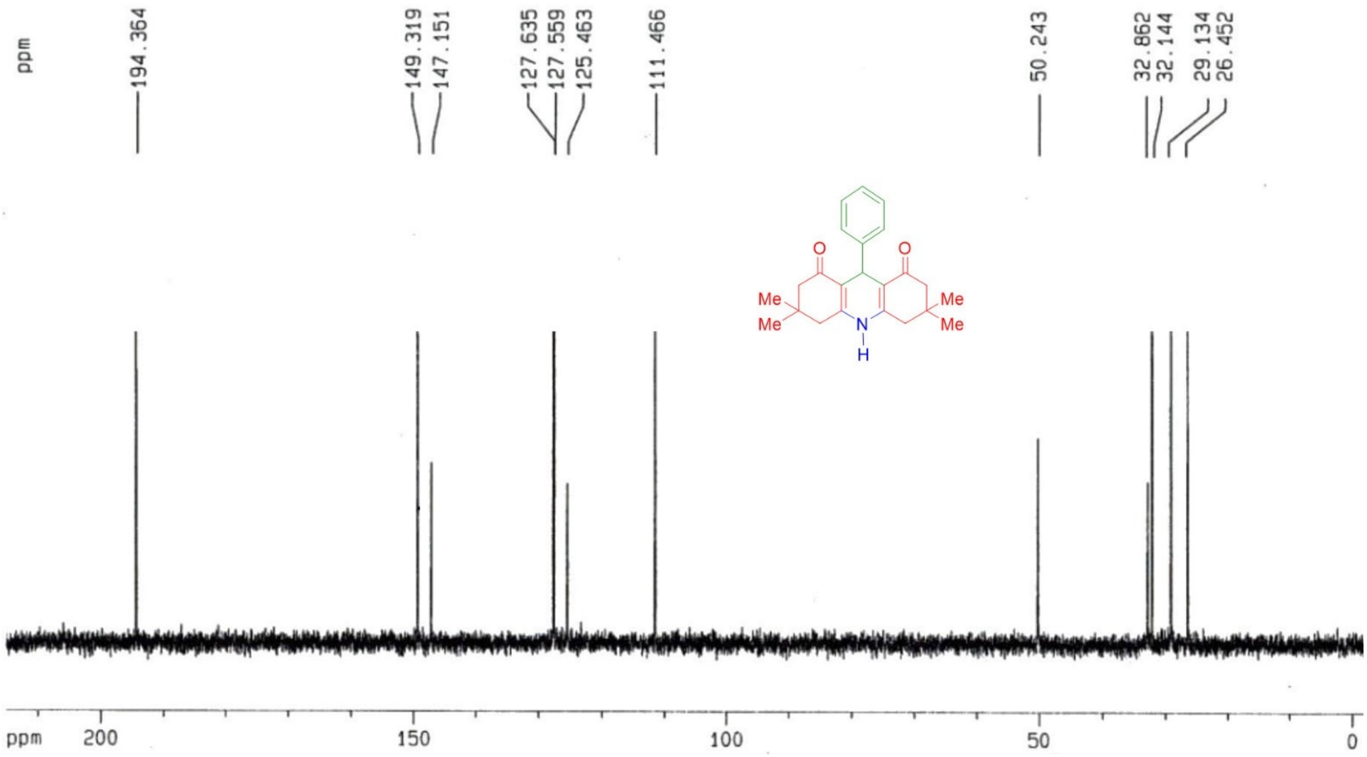

Figure S2. ^{13}C-NMR spectrum of product ***a.***

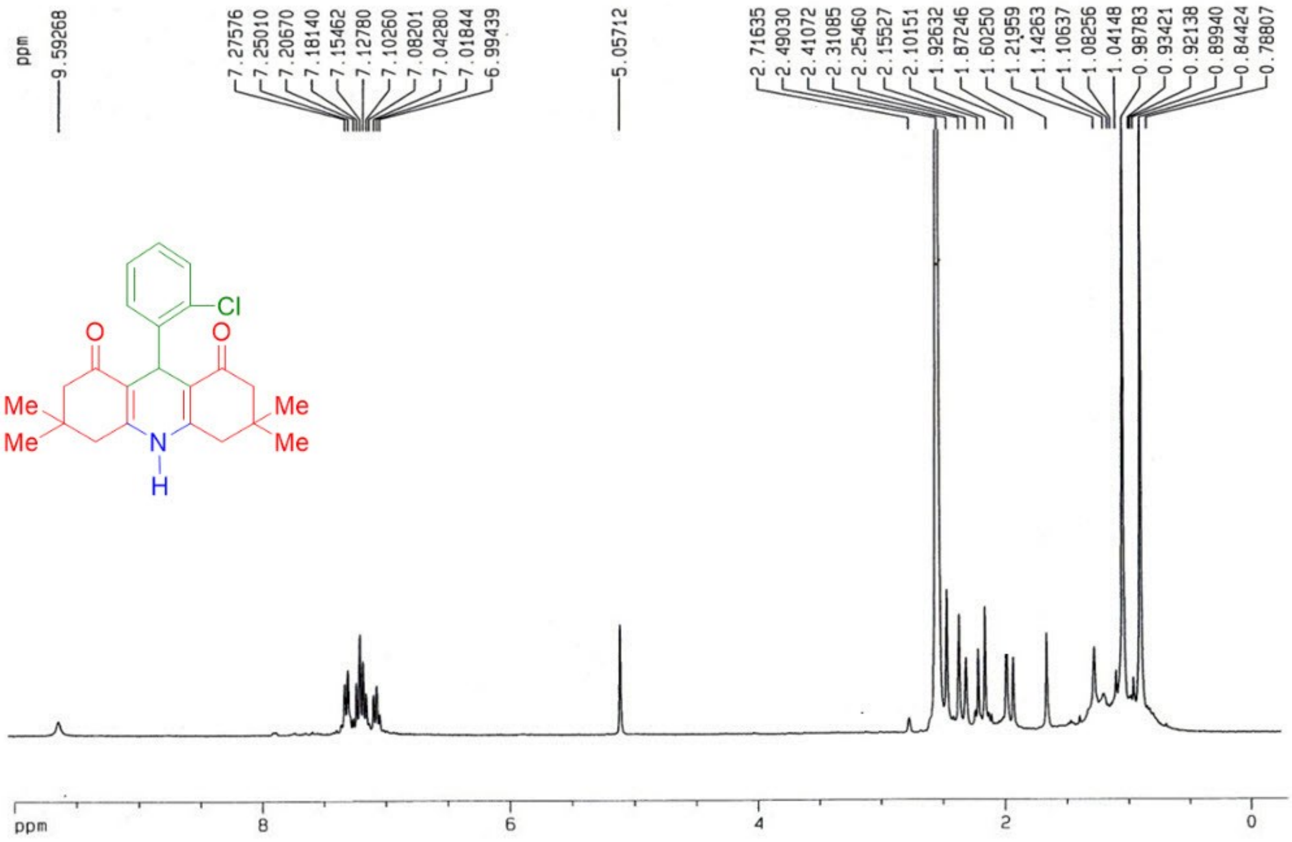

Figure S3. ^{1}H-NMR spectrum of product ***b.***

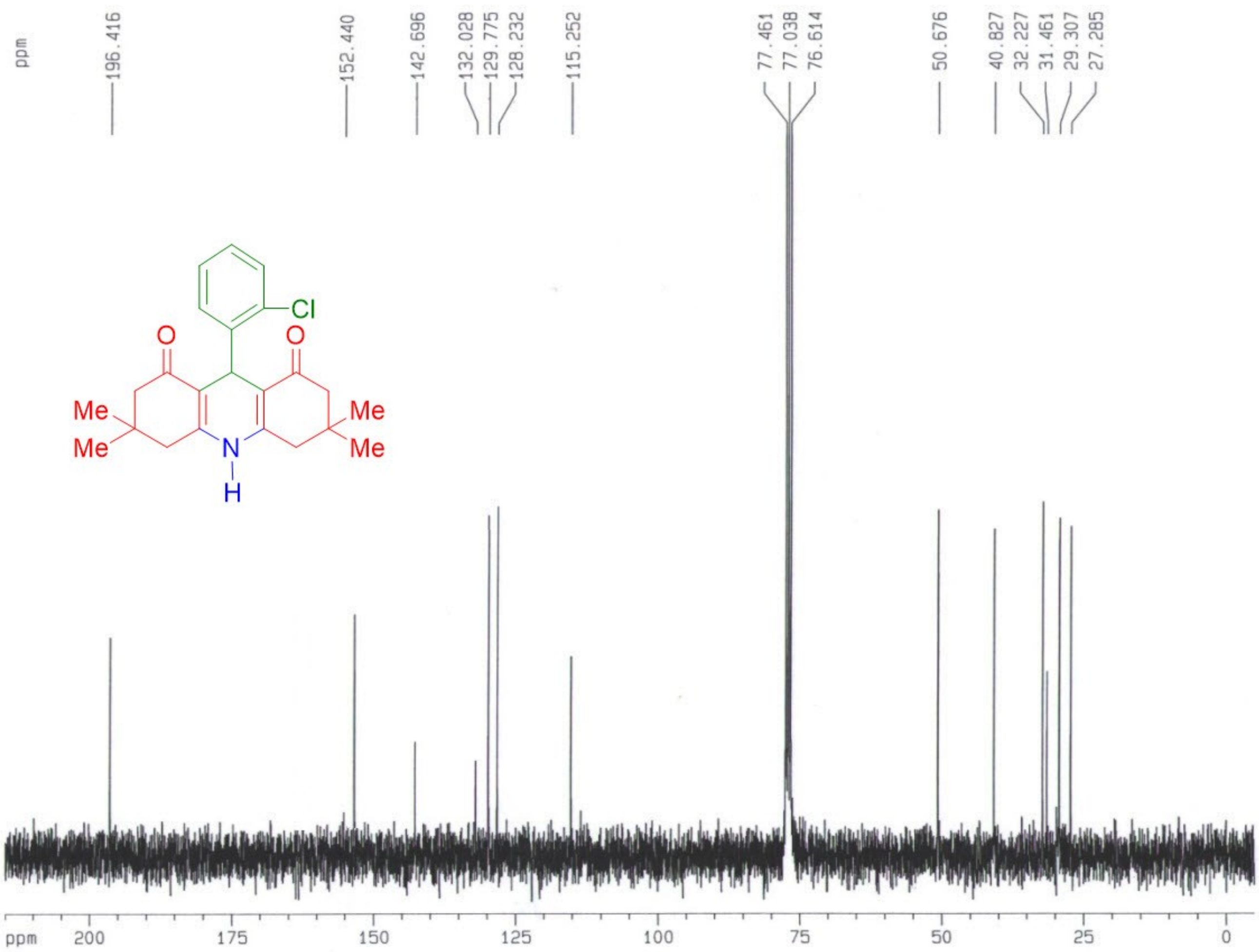

Figure S4. ^{13}C-NMR spectrum of product ***b.***

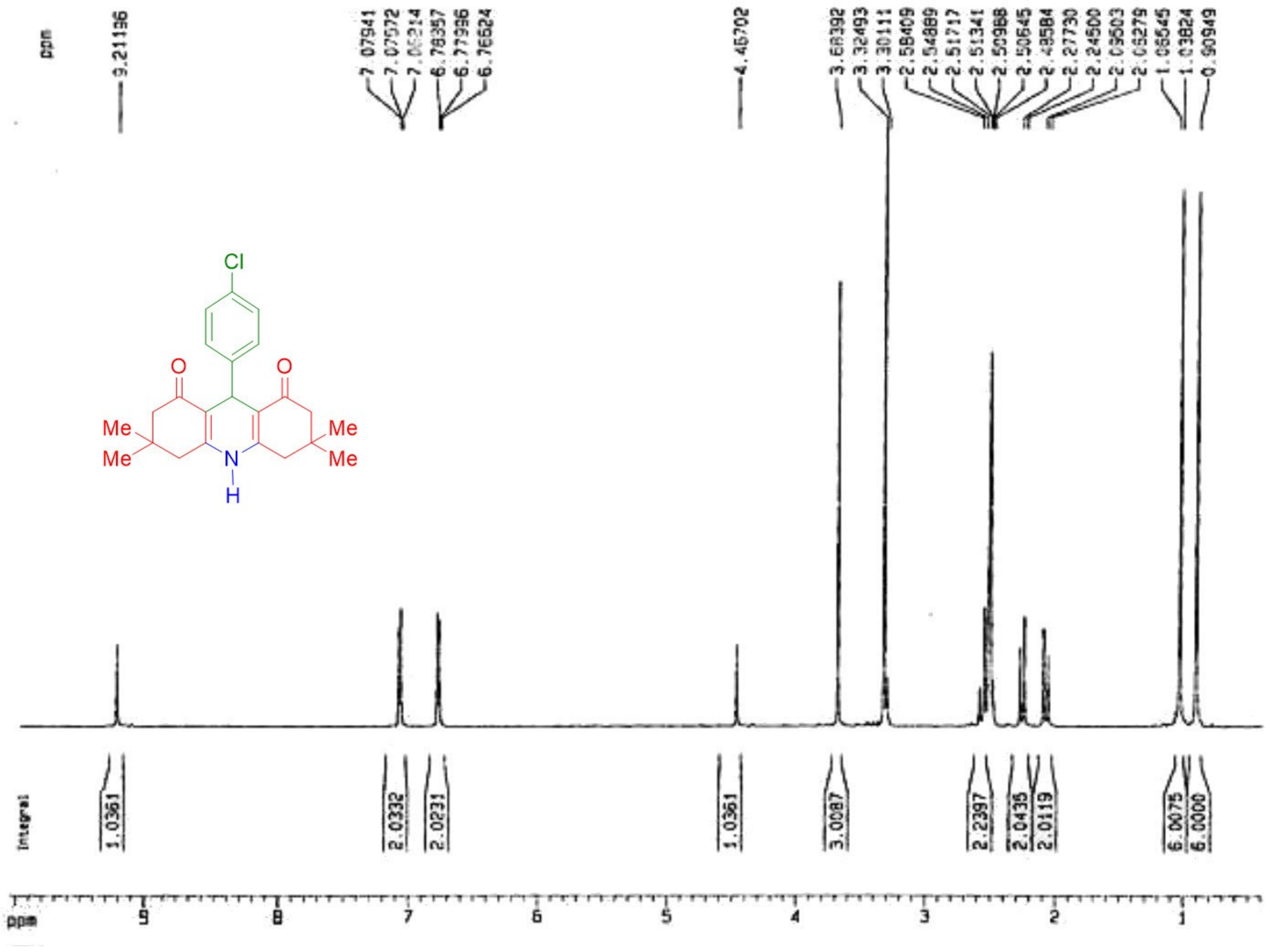

Figure S5. ^{1}H-NMR spectrum of product ***c.***

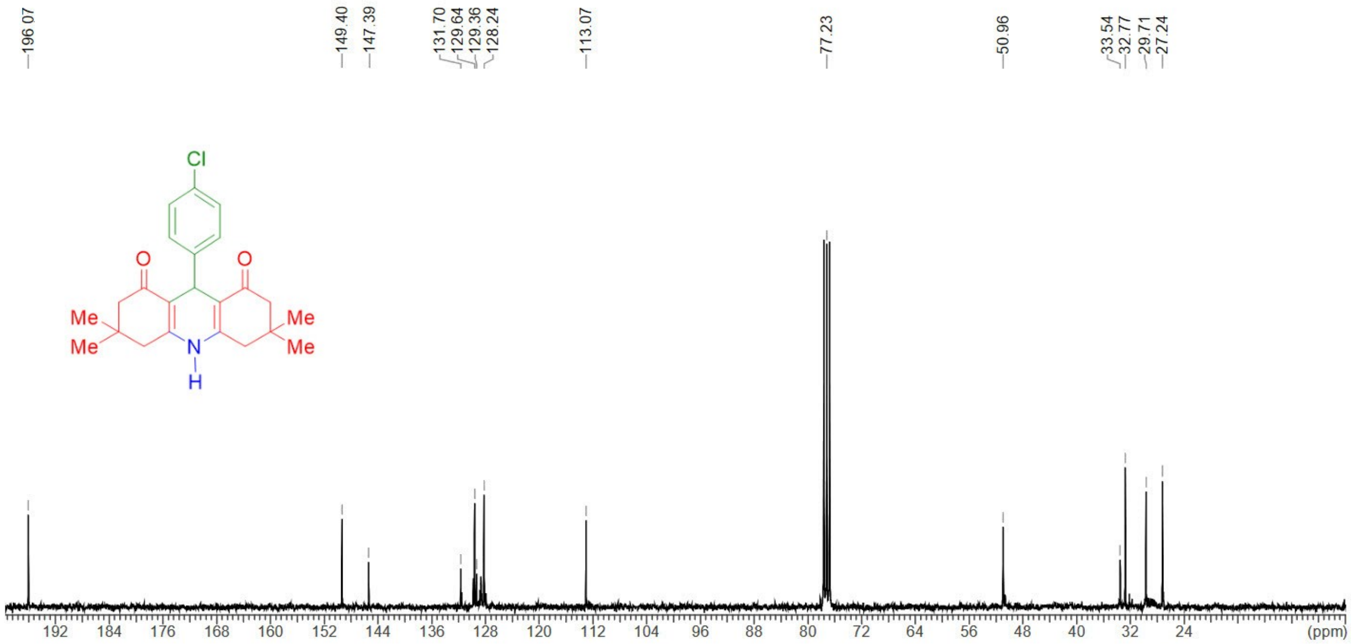

Figure S6. ^{13}C-NMR spectrum of product ***c.***

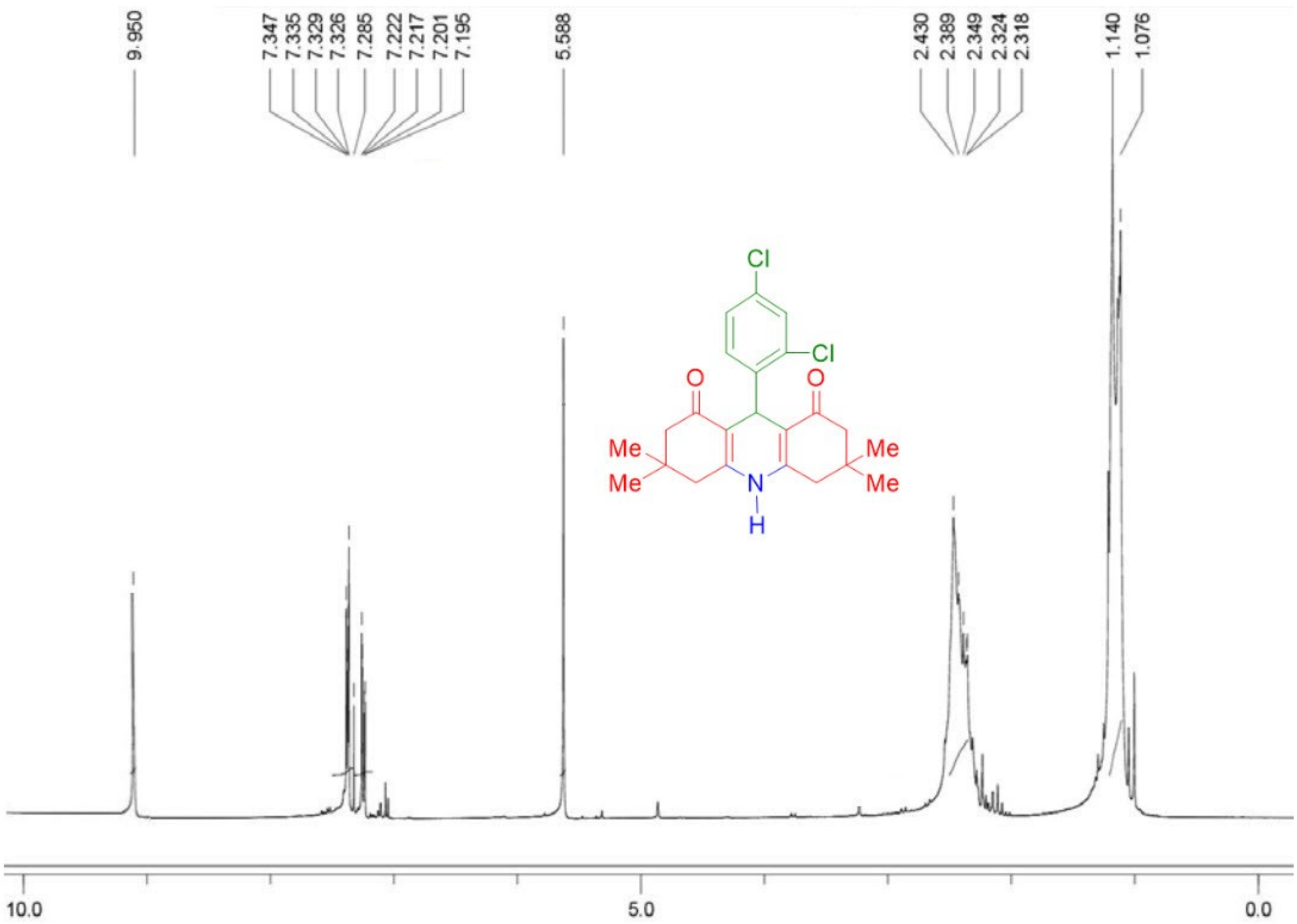

Figure S7. ^{1}H-NMR spectrum of product ***d.***

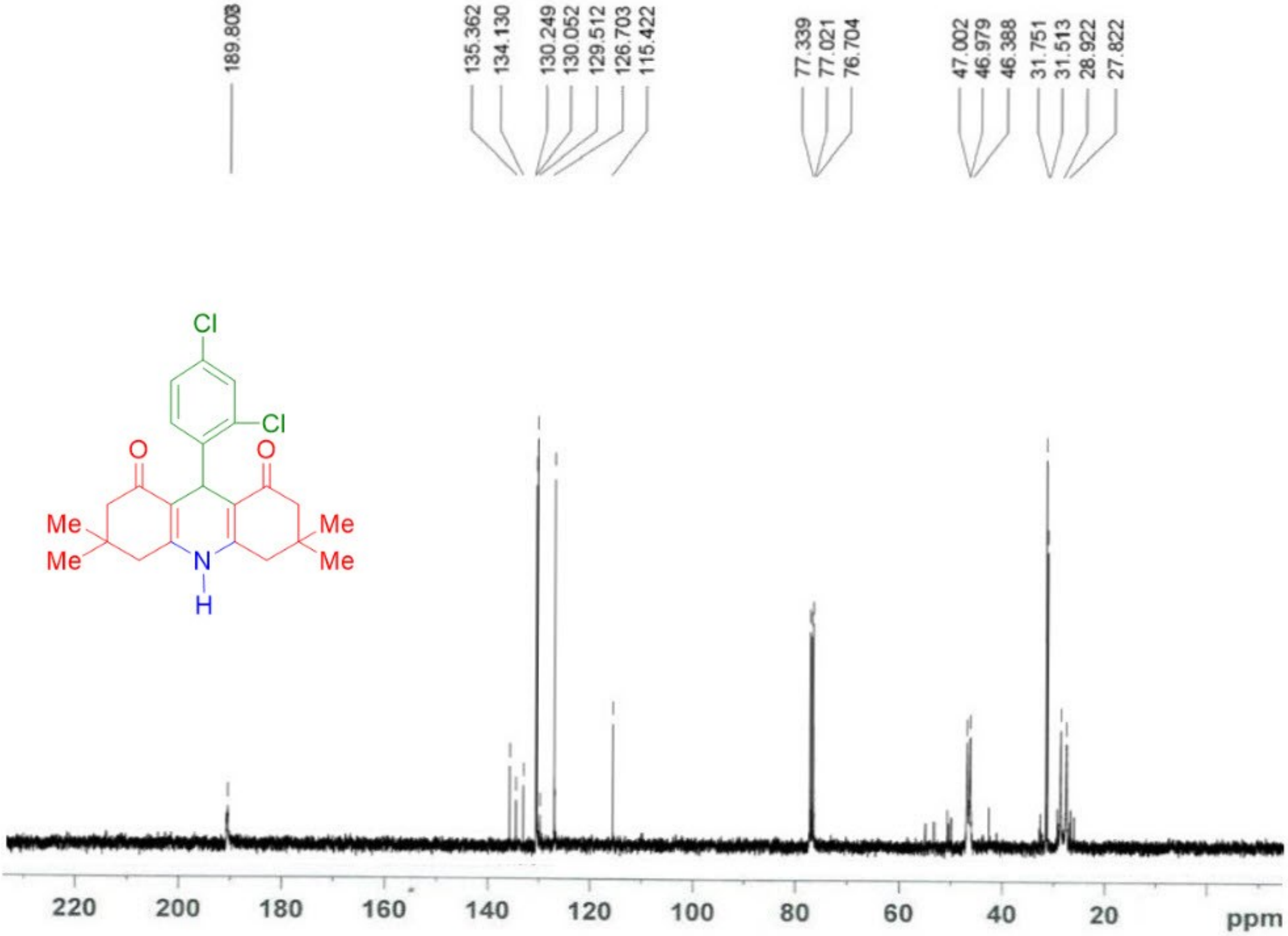

Figure S8. ^{13}C-NMR spectrum of product ***d.***

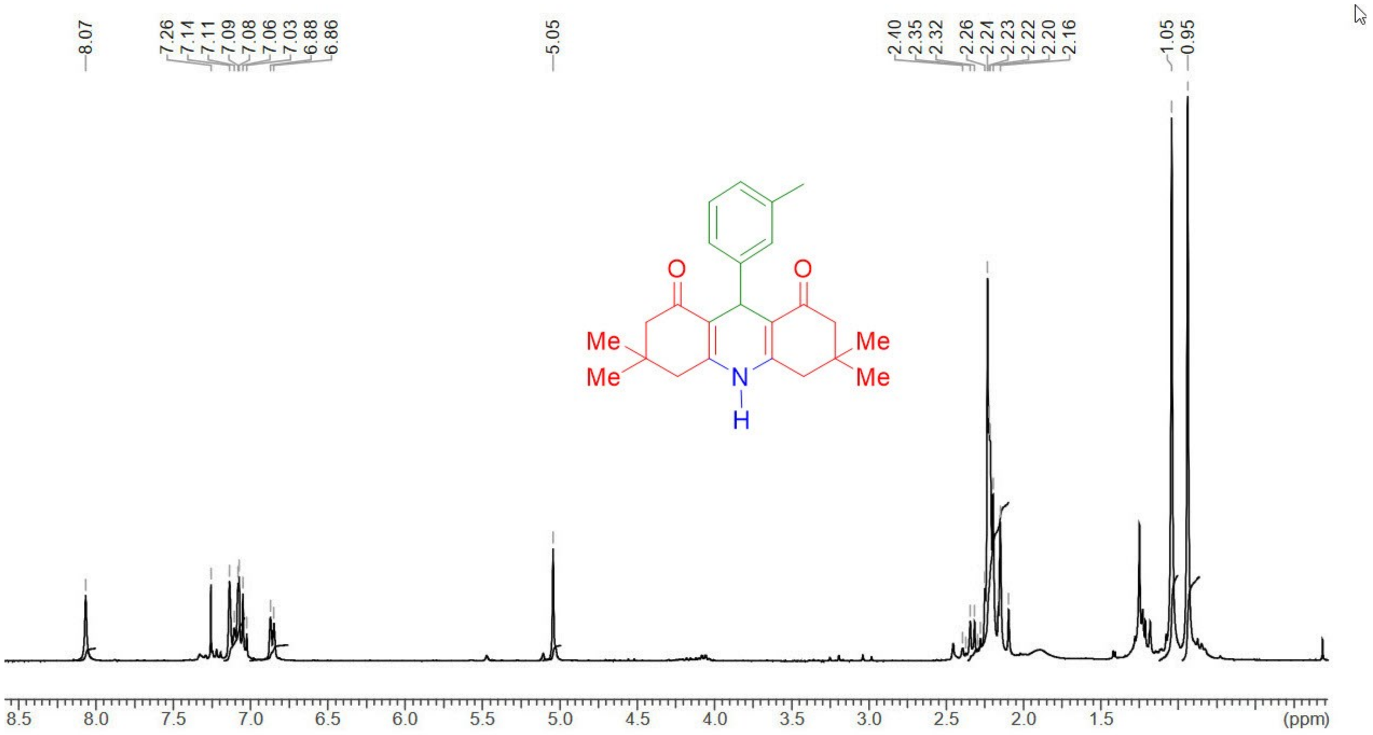

Figure S9. ^{1}H-NMR spectrum of product ***e.***

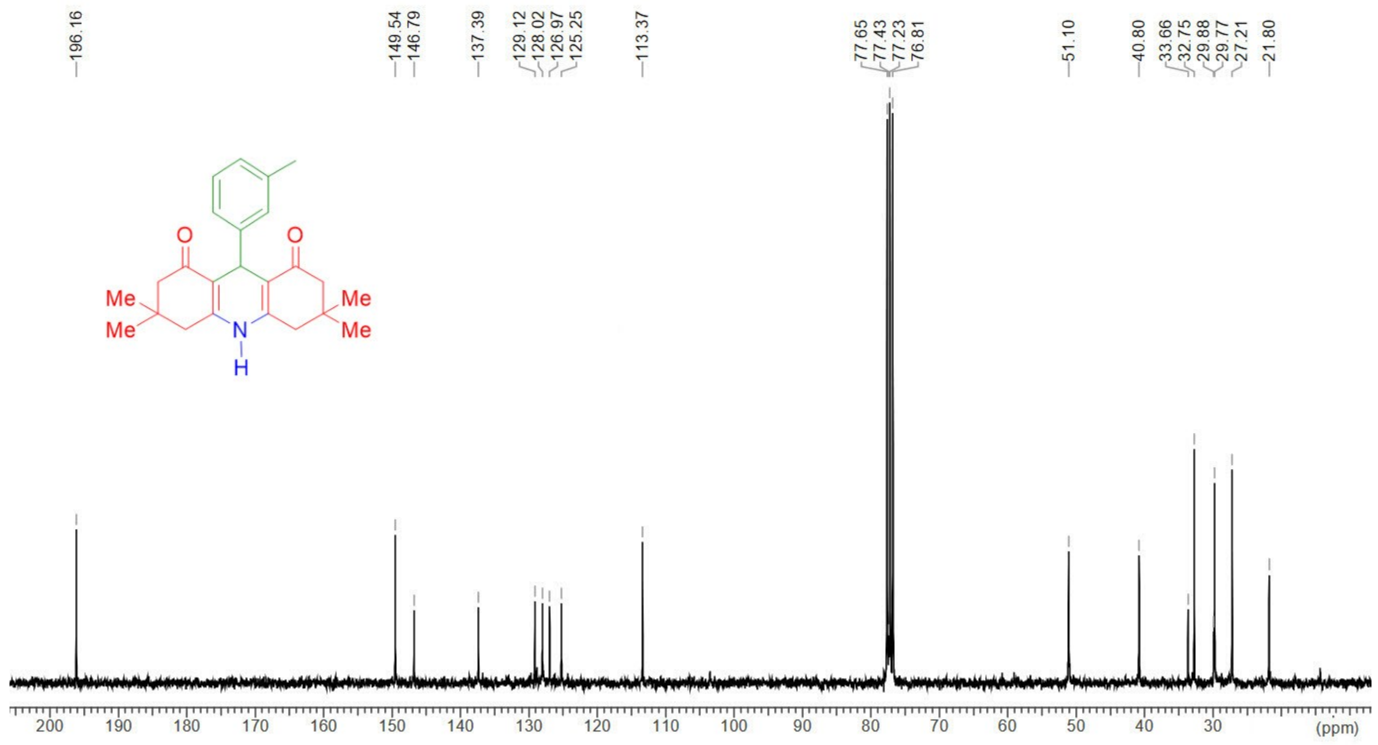

Figure S10. ^{13}C-NMR spectrum of product ***e.***

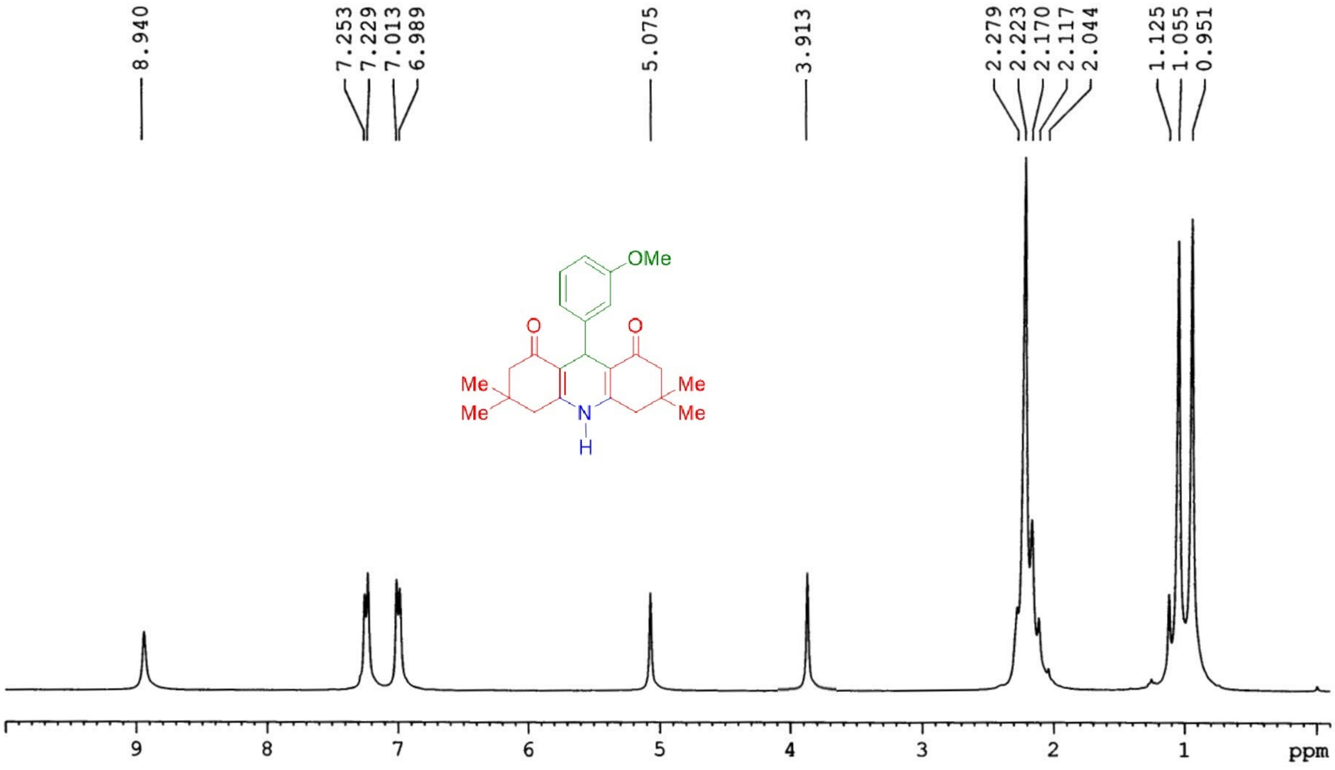

Figure S11. ^{1}H-NMR spectrum of product ***f.***

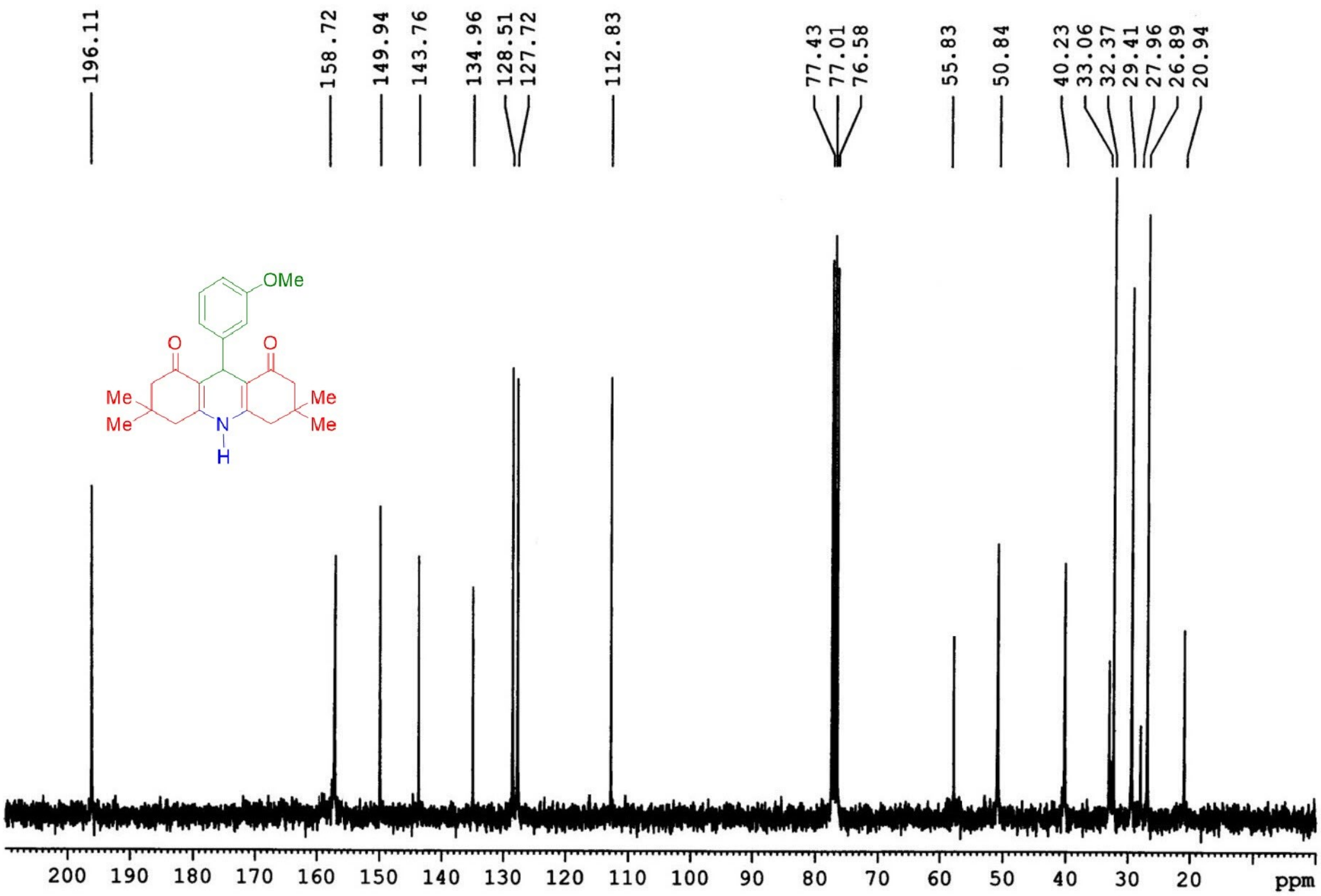

Figure S12. ^{13}C-NMR spectrum of product ***f.***

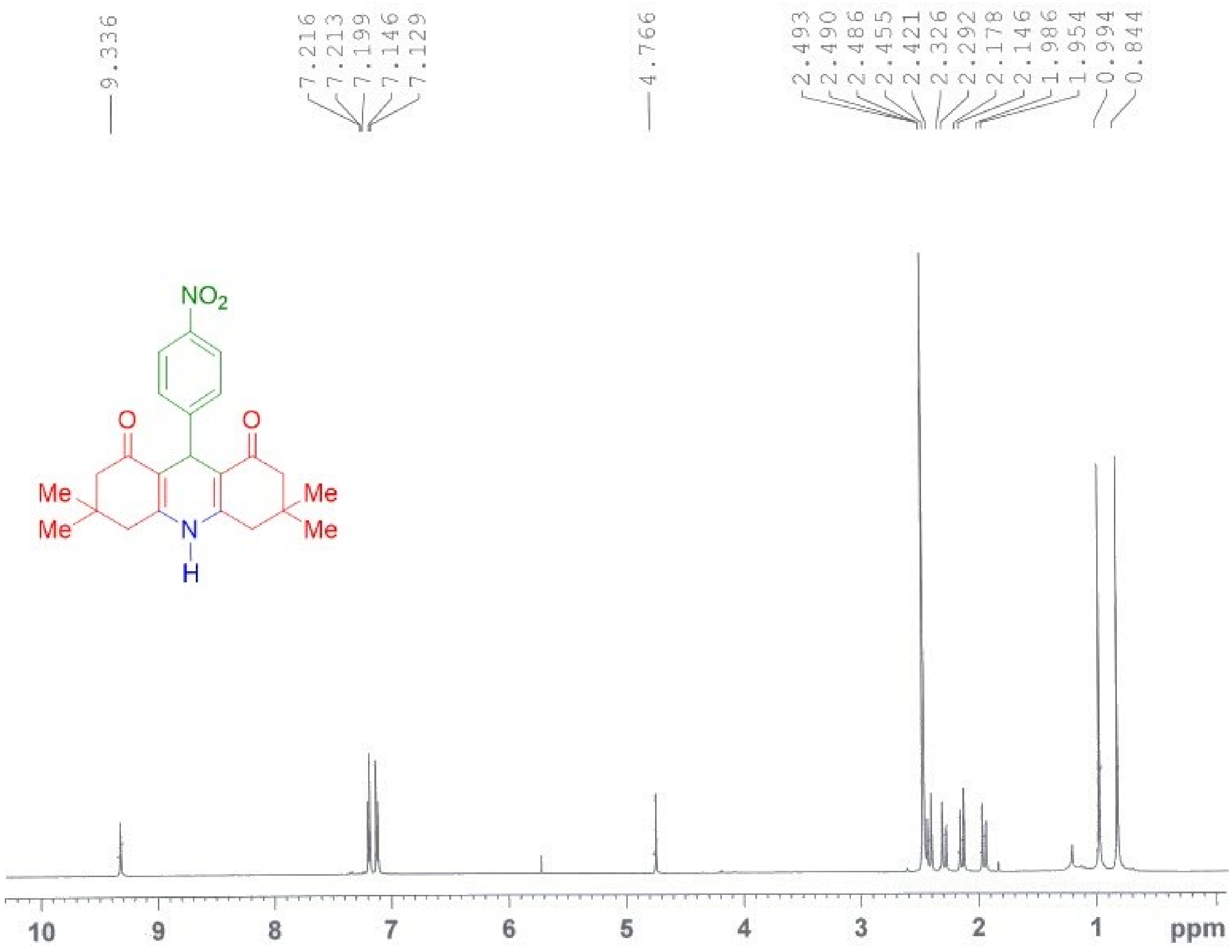

Figure S13. ^{1}H-NMR spectrum of product ***g.***

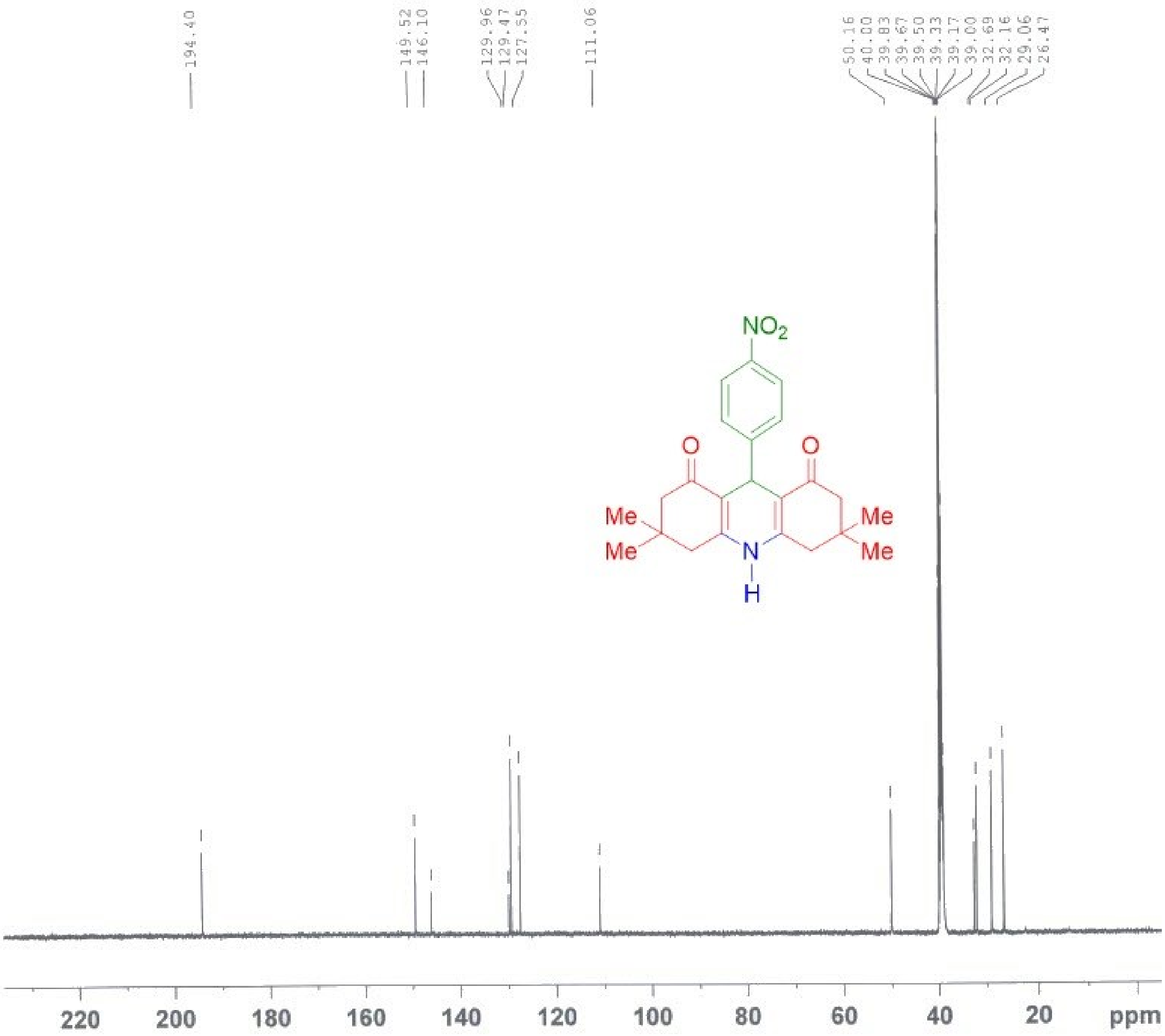

Figure S14. ^{13}C-NMR spectrum of product ***g.***

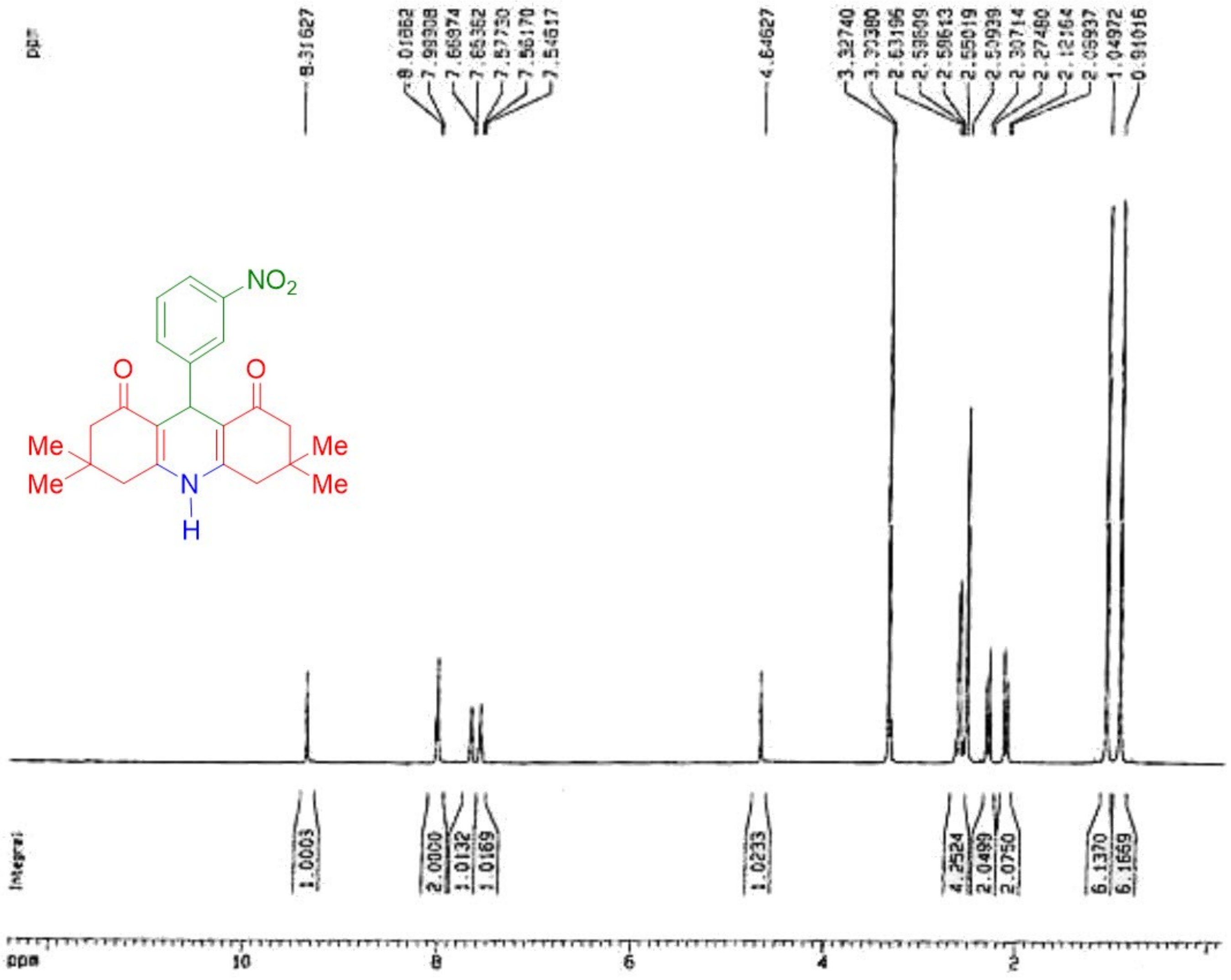

Figure S15. ^{1}H-NMR spectrum of product ***h*.**

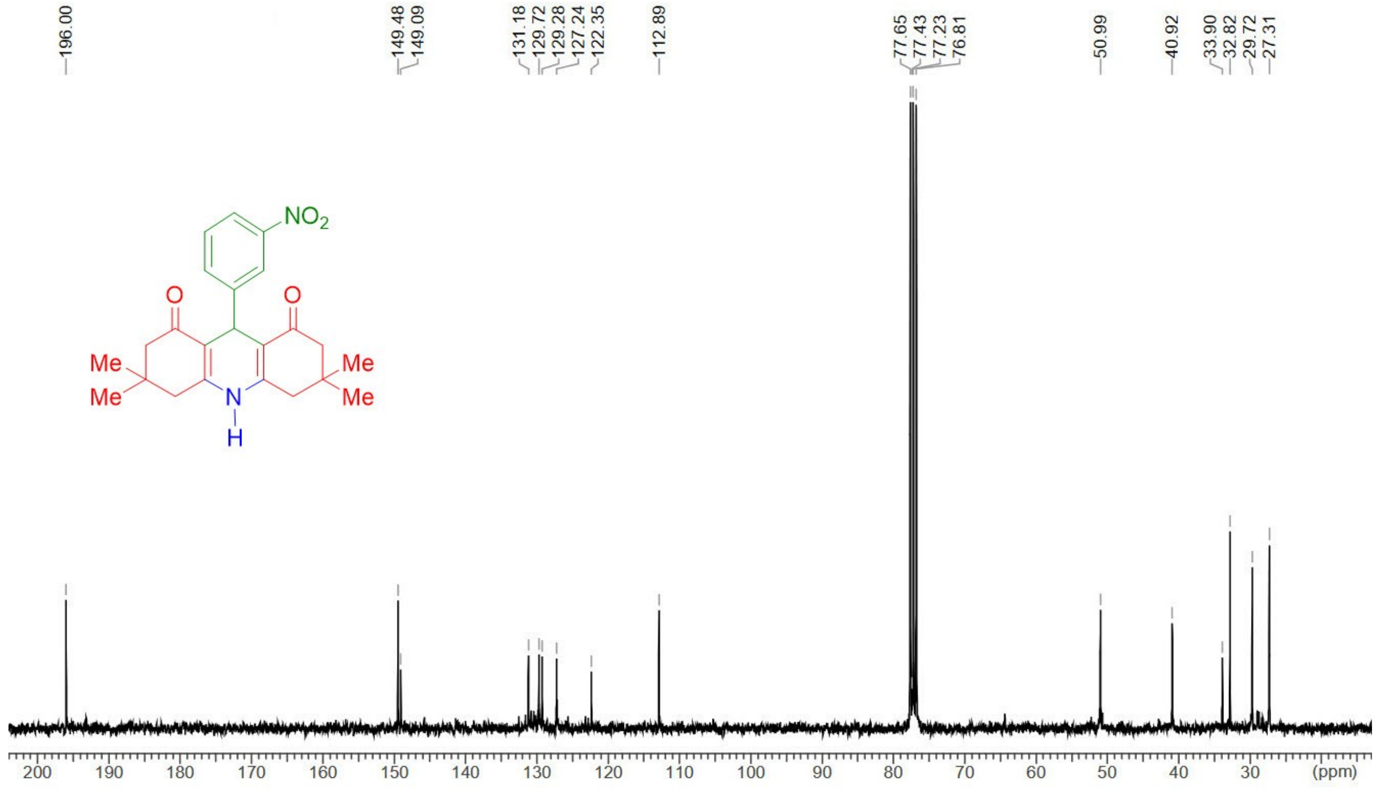

Figure S16. ^{13}C-NMR spectrum of product ***h.***

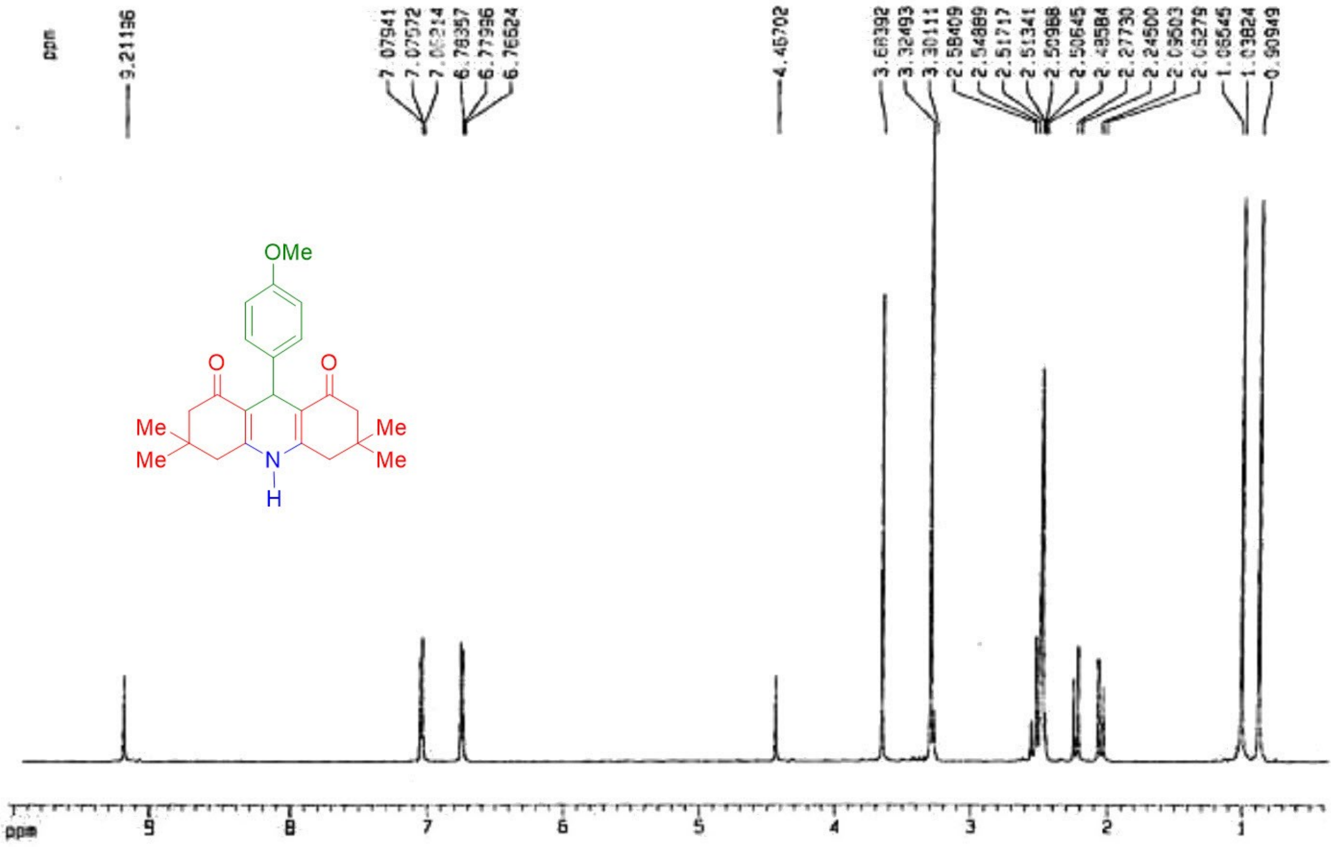

Figure S17. ^{1}H-NMR spectrum of product ***i.***

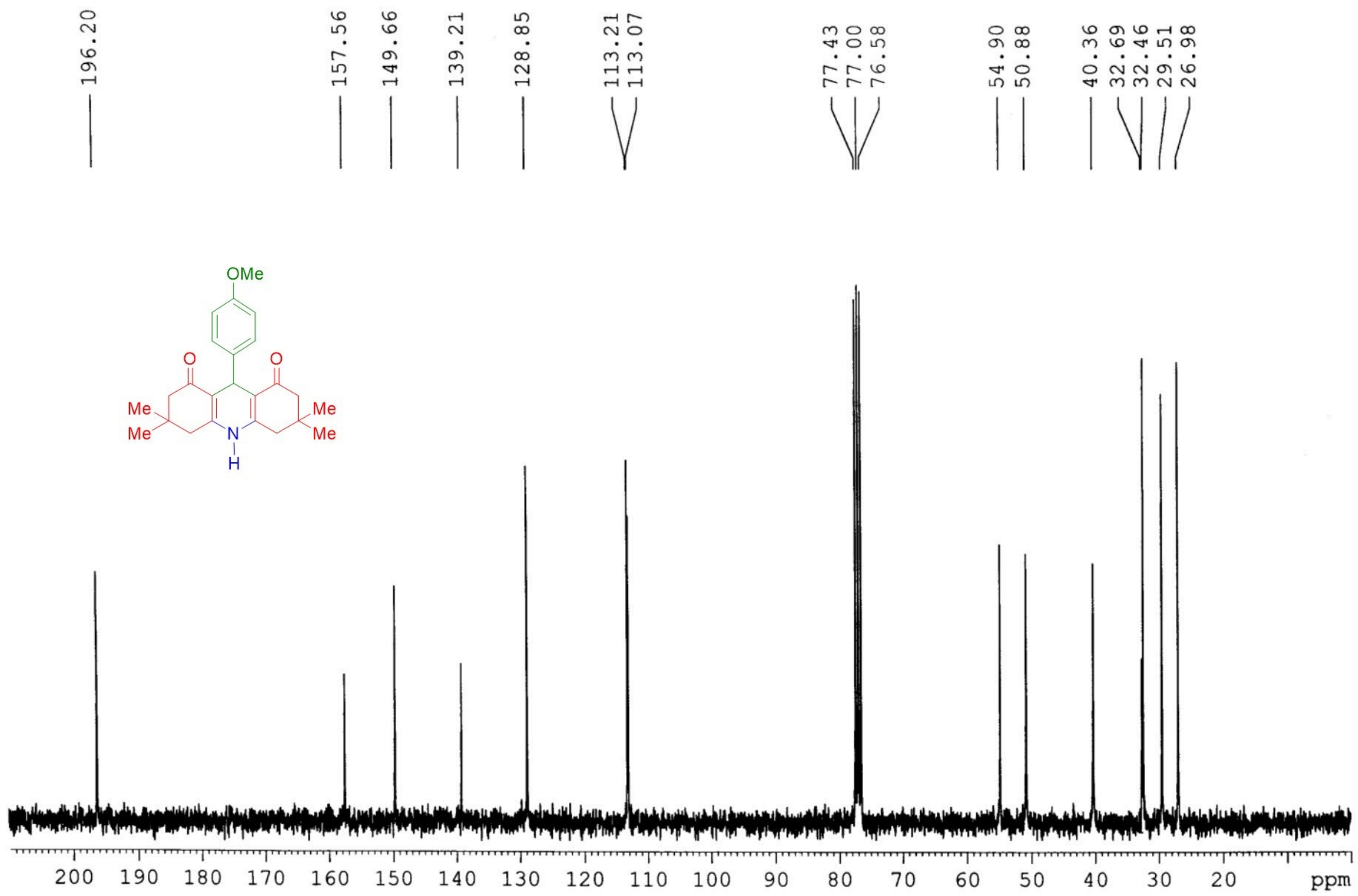

Figure S18. ^{13}C-NMR spectrum of product ***i.***

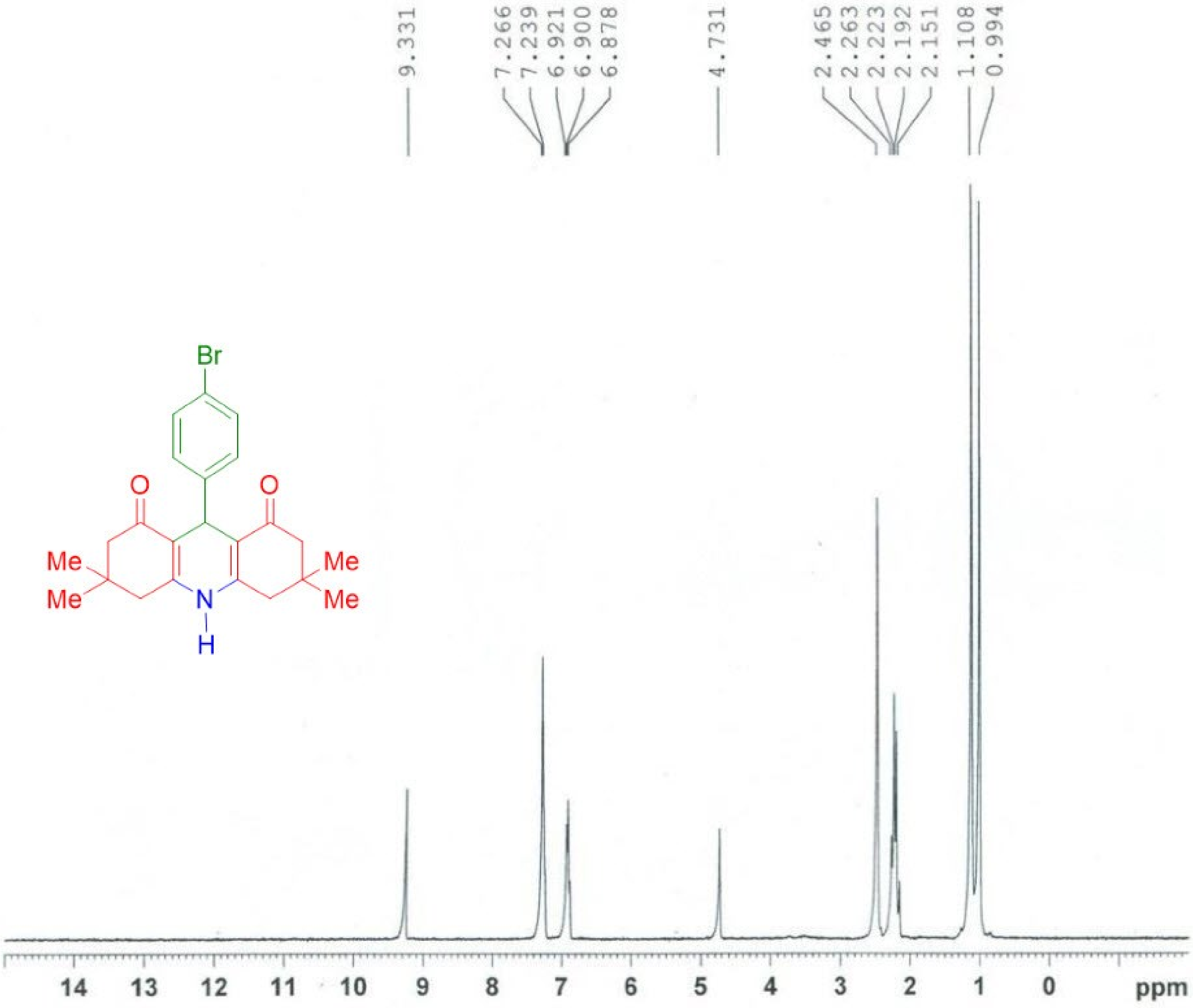

Figure S19. ^{1}H-NMR spectrum of product ***j.***

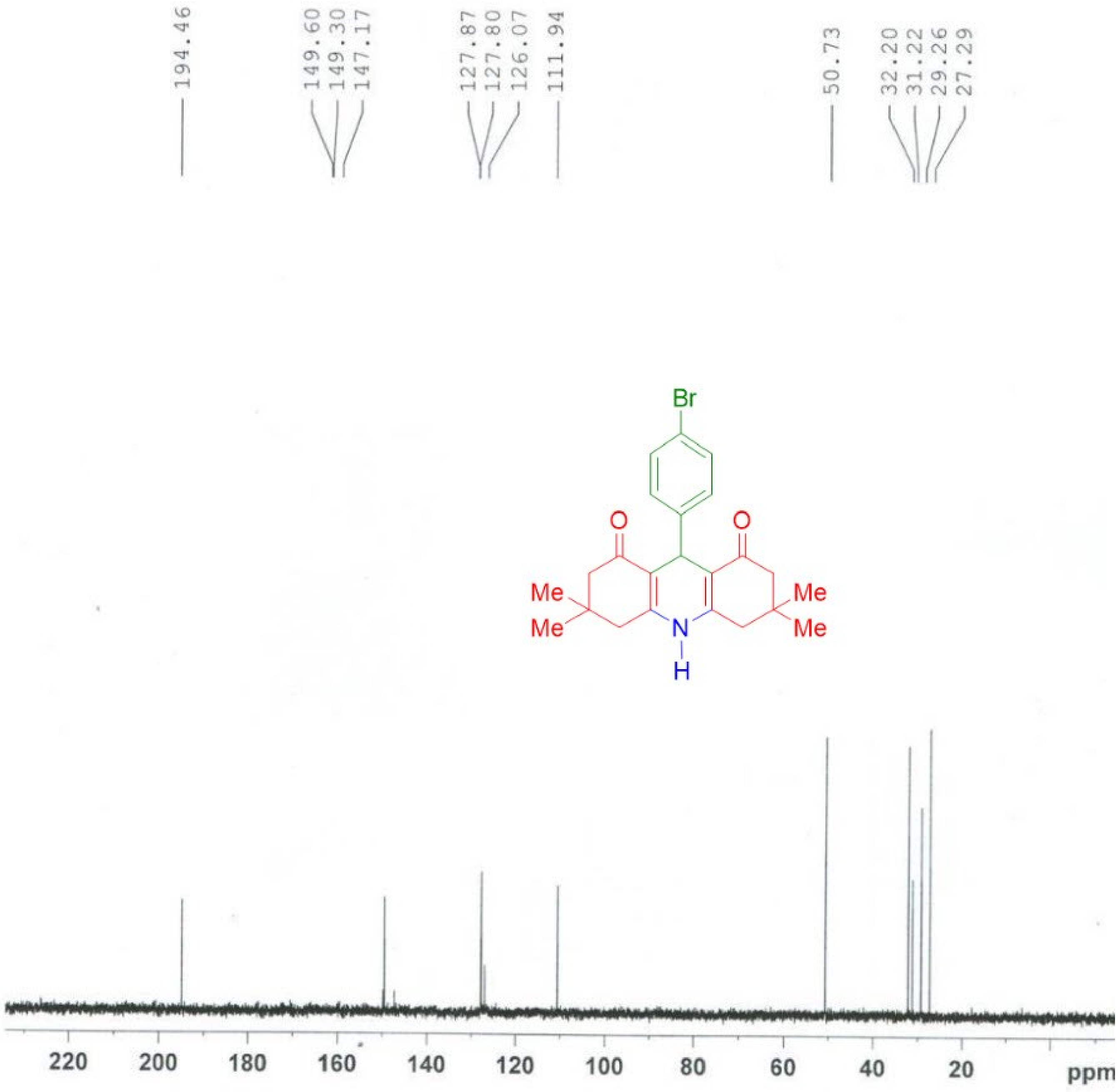

Figure S20. ^{13}C-NMR spectrum of product ***j.***
